# The baseline examinations of the German National Cohort (NAKO): recruitment protocol, response, and weighting

**DOI:** 10.1007/s10654-025-01219-8

**Published:** 2025-04-22

**Authors:** Stefan Rach, Matthias Sand, Achim Reineke, Heiko Becher, Karin Halina Greiser, Kathrin Wolf, Kerstin Wirkner, Carsten Oliver Schmidt, Sabine Schipf, Karl-Heinz Jöckel, Lilian Krist, Wolfgang Ahrens, Hermann Brenner, Stefanie Castell, Sylvia Gastell, Volker Harth, Bernd Holleczek, Till Ittermann, Stefan Janisch-Fabian, André Karch, Thomas Keil, Carolina J. Klett-Tammen, Alexander Kluttig, Oliver Kuß, Michael Leitzmann, Wolfgang Lieb, Claudia Meinke-Franze, Karin B. Michels, Rafael Mikolajczyk, Ilais Moreno Velásquez, Nadia Obi, Cara Övermöhle, Annette Peters, Tobias Pischon, Susanne Rospleszcz, Börge Schmidt, Matthias B. Schulze, Andreas Stang, Henning Teismann, Christine Töpfer, Robert Wolff, Kathrin Günther

**Affiliations:** 1 Department of Epidemiological Methods and Etiological Research, Leibniz Institute for Prevention Research and Epidemiology - BIPS, Achterstr. 30, 28359 Bremen, Germany; 2 Department of Survey Design and Methodology, GESIS—Leibniz Institute for the Social Sciences, Mannheim, Germany; 3 Department of Biometry and Data Management, Leibniz Institute for Prevention Research and Epidemiology - BIPS, Bremen, Germany; 4 Institute of Global Health, University Hospital Heidelberg, Heidelberg, Germany; 5 Division of Cancer Epidemiology, German Cancer Research Center (DKFZ), Heidelberg, Germany; 6 Institute of Epidemiology, Helmholtz Zentrum München - German Research Center for Environmental Health (GmbH), Neuherberg, Germany; 7 Leipzig Research Centre for Civilization Diseases, University of Leipzig, Leipzig, Germany; 8 Institute for Community Medicine, University Medicine Greifswald, Greifswald, Germany; 9 Medical Faculty, University of Duisburg-Essen, University Hospital Essen, Essen, Germany; 10 Institute of Social Medicine, Epidemiology and Health Economics, Charité-Universitätsmedizin Berlin, Berlin, Germany; 11 Division of Clinical Epidemiology and Aging Research, German Cancer Research Center (DKFZ), Heidelberg, Germany; 12 Department of Epidemiology, Helmholtz Centre for Infection Research (HZI), Brunswick, Germany; 13 German Institute of Human Nutrition Potsdam-Rehbruecke, Nuthetal, Germany; 14 Institute for Occupational and Maritime Medicine (ZfAM), University Medical Center Hamburg-Eppendorf, Hamburg, Germany; 15 Saarland Cancer Registry, Saarbrücken, Germany; 16 Institute of Epidemiology and Social Medicine, Münster, Germany; 17 Institute of Clinical Epidemiology and Biometry, University of Würzburg, Würzburg, Germany; 18 State Institute of Health I, Bavarian Health and Food Safety Authority, Erlangen, Germany; 19 Institute of Medical Epidemiology, Biostatistics and Informatics, Martin-Luther University Halle-Wittenberg, Halle (Saale), Germany; 20 Interdisciplinary Center for Health Sciences, Medical Faculty of the Martin-Luther-University Halle-Wittenberg, Halle (Saale), Germany; 21 German Diabetes Center (DDZ), Institute for Biometrics and Epidemiology, Düsseldorf, Germany; 22 Department of Epidemiology and Preventive Medicine, University of Regensburg, Regensburg, Germany; 23 Institute of Epidemiology, Kiel University, Kiel, Germany; 24 Institute for Prevention and Cancer Epidemiology, Faculty of Medicine and Medical Center, University of Freiburg, Freiburg, Germany; 25 Institute of Medical Epidemiology, Biostatistics, and Informatics, Medical Faculty of the Martin-Luther-University Halle-Wittenberg, Halle (Saale), Germany; 26 Molecular Epidemiology Research Group, Max-Delbrück-Center for Molecular Medicine in the Helmholtz Association (MDC), Berlin, Germany; 27 Chair of Epidemiology, Institute for Medical Information Processing, Biometry and Epidemiology, Medical Faculty, Ludwig-Maximilians-Universität München, Munich, Germany; 28 Max-Delbrück-Center for Molecular Medicine in the Helmholtz Association (MDC), Biobank Technology Platform, Berlin, Germany; 29 Corporate member of Freie Universität Berlin and Humboldt-Universität Zu Berlin, Charité - Universitätsmedizin Berlin, Berlin, Germany; 30 Department of Diagnostic and Interventional Radiology, Faculty of Medicine, University Medical Center Freiburg, University of Freiburg, Freiburg, Germany; 31 Institute for Medical Informatics, Biometry and Epidemiology, University Hospital Essen, University Duisburg-Essen, Essen, Germany; 32 Institute of Nutritional Science, University of Potsdam, Nuthetal, Germany; 33 Trusted Third Party of the University Medicine Greifswald, Greifswald, Germany

**Keywords:** Population-based, Cohort studies, Epidemiology, Response, Nonresponse, Participation, Sample design, Survey weights, Correction weights

## Abstract

The German National Cohort (NAKO) is the largest population-based epidemiologic cohort study in Germany and investigates the causes of the most common chronic diseases. Between 2014 and 2019, a total of 1.3 million residents aged 20–69 years from 16 German regions were randomly selected from the general population and invited to participate following a highly standardized recruitment protocol. The overall response was 15.6% and differed considerably across study centers (7.6–30.7%). Females were more likely to participate than males (17.5% vs. 14.1%) and participation increased with age (10.2% in age group “ < 29 years” up to 20.7% in age group “ > 60 years”). Across all study regions, response was highest in rural areas (22.3%), followed by towns and suburbs (17.2%), and was lowest in cities (14.5%). Compared with the general population in the respective study regions, participants with low and medium education are underrepresented in the NAKO sample, while highly educated participants are overrepresented. Participants with non-German nationality and with a migration background are also underrepresented. Participants living in single households are underrepresented, while participants from larger households (2 or more persons) are overrepresented compared to the general population. Survey weights are made available to researchers along with the study data that account for the sampling design and adjust for differences in the distribution of age, sex, nationality (German vs. non-German), migration status, education, and household size.

**Supplementary Information:**

The online version contains supplementary material available at 10.1007/s10654-025-01219-8.

## Background

The willingness to participate in population-based research has been declining for decades [1–5]. The causes of this decline are only partly understood [6], the true extent of the problem may be obscured by inadequate reporting [3], and strategies to reverse this trend are still unclear [7, 8]. It is known, however, that the decision to participate in health research is often associated with higher educational and social status, healthier lifestyles, and a better health status (e.g., [9]). Although it remains a matter of debate whether low response proportions inevitably impair generalizability [10–15], there seems to be a consensus that a higher response is generally preferable [14, 16].

Some consequences of differential nonresponse can be addressed by statistical weighting techniques. Weighting is the process of assigning a factor to individual study participants according to their relative importance for calculating estimates of population parameters [17]. Study participants often differ in their probability to be included in a study, which can introduce bias in prevalence estimates if not accounted for, just as nonresponse in the study can do. The aim of statistical weighting is to reduce bias in the estimates, hence to increase the resemblance between the estimated parameters in the sample and the true parameters in the study base (i.e., the underlying target population). It is important to note that, while representativeness, and therefore weighting, are important for descriptive purposes (e.g., estimates of prevalence, risk, or exposure), they are less relevant, or in certain instances, even counterproductive for the investigation of etiological research questions [11].

Here we report on the recruitment and participation in the German National Cohort (NAKO, “NAKO Gesundheitsstudie”), supplementing the previous description of the examination protocol [18]. NAKO is the largest epidemiological population-based cohort study in Germany and investigates the causes of the most common chronic diseases [19]. It was initiated by a network of 18 study centers across 16 regions in Germany as part of a collaboration of 27 German scientific institutions, including 15 universities, 4 institutions of the Helmholtz Association, 4 institutes of the Leibniz Association, and 4 other national research institutions. Baseline examinations were conducted from 2014 to 2019 and included computer-assisted personal face-to-face interviews, a series of standardized physical and medical examinations, the collection of various biomaterials, and self-administered questionnaires for the standard Level 1 program. Additional in-depth examinations were offered to 20% randomly selected participants (Level 2 program). Whole body magnetic resonance imaging was offered to more than 30.000 participants who were all also enrolled into the Level 2 program (if they weren’t already). Detailed descriptions of the study design, the baseline examination protocol, and the baseline sample have been published elsewhere [18–21].

In the following we provide a detailed description of NAKO’s sample design and recruitment protocol, a descriptive analysis of response proportions and reasons for non-participation as well as a methodological description of the survey weights that are provided together with the NAKO data set.

## Methods

### Sample design

Based on recommendations of an international expert panel, 18 NAKO study centers were selected non-randomly from applications of German research institutions (Table 1). Selection criteria included practical experience in conducting population-based prospective cohort studies, experience in using standardized assessment instruments, and a strong track record in chronic disease research.

**Table 1:** Study centers in the German National Cohort (NAKO)

		Geographical region		Recruitment area	
Study Center	Target	North/Central/South	East/West	Federal state	Urbanization^a^
Bremen	10,000	North	West	Bremen	Densely populated
Hamburg	10,000	North	West	Hamburg	Densely populated
Hannover	10,000	North	West	Lower-Saxony	Densely populated
Kiel	10,000	North	West	Schleswig–Holstein	Mixture of all three categories
Neubrandenburg^b^	20,000	North	East	Mecklenburg-Vorpommern	Predominantly thinly populated
Berlin-Mitte^c^	10,000	Berlin-Brandenburg	East	Berlin	Densely populated
Berlin-Nord^c^	10,000	Berlin-Brandenburg	East	Berlin and Brandenburg	Predominantly densely populated
Berlin-Süd^c^	10,000	Berlin-Brandenburg	East	Berlin and Brandenburg	Predominantly densely populated
Halle	10,000	Central	East	Saxony-Anhalt	Predominantly densely populated
Leipzig	10,000	Central	East	Saxony	Predominantly densely populated
Düsseldorf	10,000	Central	West	North Rhine-Westphalia	Densely populated
Essen	10,000	Central	West	North Rhine-Westphalia	Densely populated
Münster	10,000	Central	West	North Rhine-Westphalia	Densely populated
Augsburg	20,000	South	West	Bavaria	Mixture of all three categories
Freiburg	10,000	South	West	Baden-Württemberg	Mixture of all three categories
Mannheim	10,000	South	West	Baden-Württemberg	Densely populated
Regensburg	10,000	South	West	Bavaria	Mixture of all three categories
Saarbrücken	10,000	South	West	Saarland	Predominantly intermediate density

^a^Urbanization was categorized according to DEGURBA [23] into cities (densely populated areas), towns and suburbs (intermediate density areas), and rural areas (thinly populated areas)

^b^Neubrandenburg operated one permanent examination center in the city of Neubrandenburg and temporary examination centers in Neustrelitz (May 2014—April 2016), Waren an der Müritz (May 2016—June 2017), and Demmin (July 2017—April 2018)

^c^The city of Berlin was divided into three separate areas, each of which was managed by one of three study centers in Berlin-Mitte, Berlin-Nord and Berlin-Süd

Study regions in the catchment areas of these study centers were shaped such that the NAKO source population achieved an appropriate balance with respect to regional distribution (South/Central/North and East/West Germany), rural versus urban areas, and variation in regional indicators of socioeconomic status (unemployment rate, poverty risk). A minimum of 10,000 participants per study center was considered necessary for reasons of cost-effectiveness, standardization of examination procedures, and quality control of the data collection. The two centers with the most extensive existing infrastructure for cohort studies—Augsburg and Neubrandenburg—were selected to act as double-recruitment centers, recruiting 20.000 participants each.

The planned regional distribution was as follows: 60,000 participants in the northern study area (study centers Bremen, Hamburg, Hannover, Kiel, Neubrandenburg), 30,000 participants in the metropolitan region of Berlin-Brandenburg (Berlin-Nord, Berlin-Mitte, Berlin-Süd), 50,000 participants in the central study area (Halle, Leipzig, Düsseldorf, Essen, Münster), and 60,000 participants in the southern study area (Augsburg, Freiburg, Mannheim, Regensburg, Saarbrücken). With this distribution, 35% of the cohort would be recruited in the eastern areas (former German Democratic Republic including Berlin), resulting in an oversampling as compared to the western areas (underlying population: 20% east versus 80% west). Approximately 35% of the cohort would be recruited in densely populated areas (large cities), 30% in areas of intermediate density (400 to 2,000 inhabitants/km^2^), and 35% in rural/thinly populated areas (less than 400 inhabitants/km^2^).

The metropolitan region of Berlin-Brandenburg (city of Berlin and parts of the Federal State of Brandenburg) was divided into three separate non-overlapping areas, each of which was managed by one of three study centers (Berlin-Mitte, Berlin-Nord and Berlin-Süd), resulting in a total of 16 NAKO study regions being served by 18 study centers. Results are reported for the three individual study centers in Berlin separately rather than for the region of Berlin unless otherwise noted. The study center Neubrandenburg, which covered a large rural area in Mecklenburg-Vorpommern, operated one permanent examination center in the city of Neubrandenburg and temporary examination centers in Neustrelitz (May 2014–April 2016), Waren an der Müritz (May 2016—June 2017), and Demmin (July 2017—April 2018).

The recruitment target was to examine a total of 200,000 participants, divided into subsamples of 10,000 participants in each of 16 study centers and 20,000 participants in each of 2 larger centers (Augsburg and Neubrandenburg). The study base consisted of all persons in the age range 20 – 69 years (age at the time of sampling) residing within predefined study regions in the catchment areas of the study centers. Persons who lived in the predefined study regions at the time of sampling, but had moved out by the time of contact, could still participate in the study. German citizenship was not required for participation, but German language skills (or a translator provided by the participant) were necessary to complete written informed consent, questionnaires and examinations. Regional samples stratified by age and sex were recruited in each study center. The intended age distribution was 10% of participants in each of the 10-year age groups between 20 and 39 years, and 26.7% in each 10-year age group between 40 and 69 years, with an equal proportion of females and males in each age group. The intended age distribution was informed by statistical power calculations and the expected numbers of cases for major chronic diseases and their associated premature mortality (see [19] for a detailed discussion). A higher proportion of participants in age groups above 40 years was included because the incidence of most chronic diseases peaks beyond this age. Age groups below 40 years of age were included to allow for the study of risk factors, etiology, and possible modes for early diagnosis of chronic diseases during early adulthood.

The study centers requested random samples of the general population aged 20–69 years in their respective study regions from their local civil registration offices. Study centers independently determined the number and size of successive random samples to be drawn and, if necessary, adjusted the age and sex stratification of each sample depending on their local age- and sex-specific response and their recruitment progress. Samples subsequently drawn from the same municipality were screened for duplicates before being committed to recruitment. Information provided by the registration offices included name, address, sex assigned at birth (male or female), either date of birth or year of birth, and nationality. Study centers were encouraged to query publicly available or commercial telephone directories for landline and mobile phone numbers of potential participants drawn into the random sample.

### Recruitment protocol

Recruitment was conducted in all 18 study centers according to a standardized protocol laid down in a standard operating procedure (SOP). Field staff was trained locally as well as in centrally organized workshops and quality was monitored with regular site visits both, by an internal quality control team and by an independent external control team maintained by the Robert Koch-Institute, Berlin [18, 19].

Recruitment always started with a postal invitation consisting of an invitation letter, a standardized leaflet informing about the NAKO study, a return form, and a stamped return envelope addressed to the study center. Study centers were encouraged to slightly adapt the invitation to local characteristics of the study center and to include letters of recommendation by local authorities or celebrities. Interested recipients could either return the form with their contact information or call their study center. If no response was received, two sequential reminder letters were sent separated by recommended waiting periods of 14 days. For potential participants whose telephone numbers were available, up to five telephone contact attempts were made before postal reminders were sent. The final step of the recruitment protocol was an invitation letter titled “Your last chance to participate” which also included a non-responder questionnaire and a stamped return envelope addressed to the study center. A non-responder questionnaire was also offered to invited persons with whom contact could be established but who declined to participate in the study.

Study centers were free to implement additional non-mandatory recruitment steps to increase response, which could include sending out a third reminder letter, attempting additional phone calls, carrying out home visits, or offering monetary and non-monetary incentives. Most centers offered compensation towards the cost of public transport to the study center or parking fees. In some centers the invitation included letters addressed to participants’ employers encouraging them to grant participants paid leave on the day of the examination.

### Response calculation

The standard definitions of the American Association for Public Opinion Research (AAPOR, [22]) distinguish four broad response categories: eligible participants, eligible non-participants, ineligible non-participants, and those of unknown eligibility. Eligible non-participants include persons who declined to participate, were not able to participate (e.g., absence due to travel or hospitalization), or never responded to invitations (non-contacts). Reasons for ineligibility included not living in the study region anymore at the time of contact, being deceased at the time of contact, or not speaking the German language sufficiently while lacking access to a translator (interviews/examinations were conducted in German). At the end of the recruitment period, ineligibility was also attested if potential participants were never invited because they belonged to an age-sex-stratum for which the quota was already met. Unknown eligibility was attested if the domestic postal operator returned the invitation letter unopened with the return codes “Moved, left no address” or “Undeliverable”.

Response proportions were calculated according to AAPOR’s most conservative response proportion (RR1, 22), which excludes ineligible non-participants from the denominator, resulting in:

$$r=\frac{P}{P+{NP}_{eligible}+{NP}_{unknown\,\,\,eligibilty}}\times 100$$

where *P* indicates the number of successfully recruited participants, *NP*_*eligible*_ comprises all eligible non-participants, and *NP*_*unkown eligibility*_ those of unknown eligibility.

### Urbanization

Urbanization was classified according to the Degree of urbanization (DEGURBA, [23]) into three categories: cities (densely populated areas), towns and suburbs (intermediate density areas), and rural areas (thinly populated areas).

### Collection of paradata

In all study centers recruitment was controlled and documented with MODYS (Modular control & documentation system for field studies, [24]), a dedicated software for epidemiological field studies. MODYS schedules predefined recruitment tasks and provides a mail merge system to generate and print study invitations and letters. All actions by field staff (e.g., interactions with potential participants, issuing of dropout codes) are logged and time-stamped by the system. Furthermore, MODYS electronically logs study paradata [25], that is, detailed data about the recruitment process itself (e.g., attempted and successful contacts with potential participants by letter, mail, or phone). Paradata used in the current report to quantify the frequency of non-mandatory recruitment steps include the number of reminder letters routinely sent out, percentage of potential participants with phone numbers available prior to the start of recruitment, and percentage of persons for which outbound call attempts were documented prior to any active response after sending out the invitation letter. Note that outbound call attempts documented after the first active response of invited persons were disregarded for this analysis, because almost all persons who signaled their interest in participation by returning the contact form were called up by field staff afterwards.

### Calculation of survey weights

Survey weights were determined in a two-step procedure (see Supplementary methods M1 for a detailed description of the weighting procedure). First, design weights were calculated to correct for unequal inclusion probabilities of individual participants of the study using the Horvitz-Thompson-Estimator [26], defined as the inverse of the inclusion probability. Using official population data from the intercensal population updates provided by the Federal Statistical Office [27] for the years 2014 to 2019, sex and age-group specific inclusion probabilities were calculated separately for each municipality covered by the study regions. In a second step calibration weights were calculated to account for differential nonresponse and to reduce the bias and variance of the estimated parameters. Variables used for calibration were age-group, sex, nationality (German vs. non-German), education (low: ISCED97 1–2, vs. medium: ISCED97 3–4 vs. high: ISCED97 5–6), migration background (yes vs. no), and household size (1 vs. 2 vs. ≥ 3 persons). Missing values in the calibration variables were imputed using the MICE algorithm [28]. Data from the official German Microzensus [27] were used to determine marginal distributions of these variables in the general population aged between 20–69 years for each administrative district included in the study regions. Using these marginal distributions, calibration weights were calculated by iterative proportional fitting (“raking”) [29] separately for each administrative district. Survey weights were obtained by multiplying design and calibration weights. Finally, survey weights were trimmed to the 1st and 99th percentile to lower the variance of the weights and reduce the influence of outliers. Survey weights are available for the whole sample as well as for the subsample completing the in-depth examinations (Level 2 program) and the subsample completing magnetic resonance imaging. For the visual comparison between the unweighted and the weighted sample the absolute frequencies per category and the sum of the survey weights per category were plotted in grouped bar charts for the variables sex, age group, nationality, migration background, household size, and education.

## Results

### Recruitment and common reasons for non-participation

**Fig. 1:**
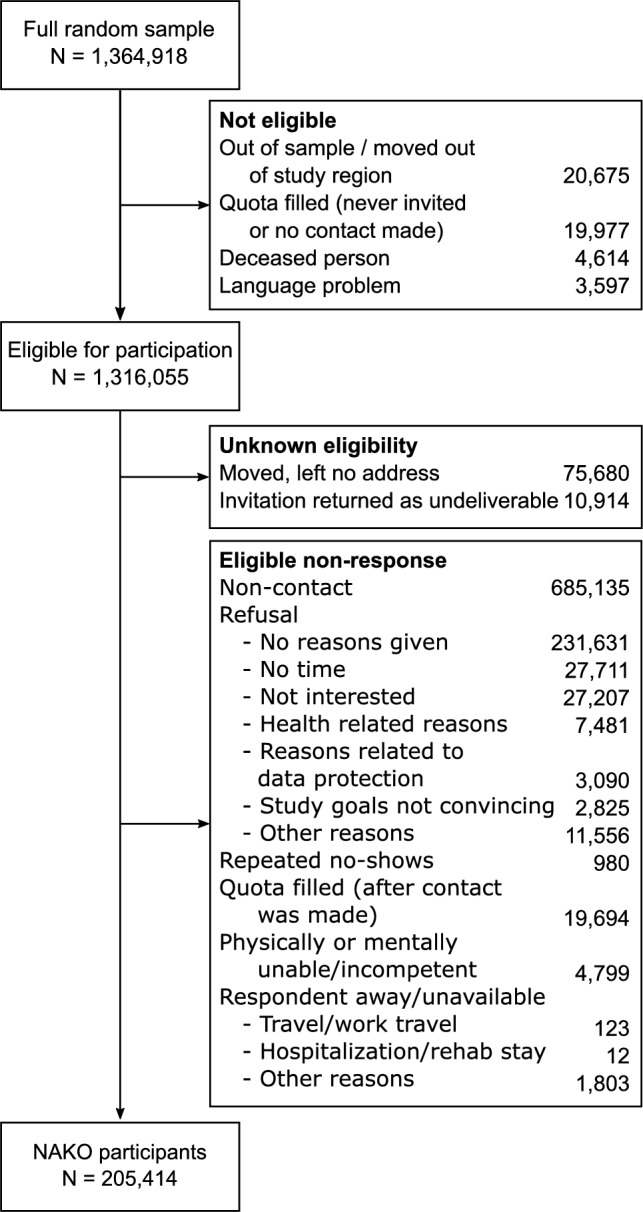
STROBE flow chart and reasons for non-participation

During the recruitment period from 2014 to 2019 (see Fig. 1 for a STROBE flow chart), a total of 1,364,918 individuals were randomly drawn from the general population of the study regions (after correction for duplicate drawings). Of these, 48,863 individuals (3.58%^1^) were not eligible for study participation, because they did not live in the study regions anymore at the time of contact (*n* = 20,675; 1.51%), were already deceased (*n* = 4,614; 0.34%), did not speak the German language and had no access to a translator (*n* = 3,597; 0.26%), or the quota of their respective age-sex-stratum was already filled before they were invited or had the chance to respond to the invitation letter (*n* = 19,977, 1.46%). Out of the remaining sample of 1,316,055 individuals, the eligibility of 86,594 individuals (6.34%) could not be determined. Either they had, at the time of invitation, moved without leaving a new address (*n* = 75,680; 5.54%), or the invitation letters were returned as undeliverable by the postal service (*n* = 10,914; 0.80%) and a validation attempt at the local civil registration offices did not provide a new address. A total of 1,024,047 individuals (75.03%) were eligible for participation but did not participate. Of these, 685,135 (50.20%) never responded to the invitation (non-contact) and 311,501 (22.82%) refused participation for various reasons (see Fig. 1 for a detailed breakdown of reasons for non-participation). Other, less common reasons for non-participation included having either insufficient physical or mental competencies for participation (*n* = 4,799; 0.35%), being unable to take part because of an absence during the study period (travel, hospitalization, or other reasons; *n* = 1,938; 0.14%), or repeatedly failing to show-up for the examination (*n* = 980; 0.07%). Finally, 19,694 individuals (1.44%) had replied and expressed interest to participate but their respective age-sex-stratum was filled before they could enroll into the study. The final study sample consisted of 205.414 (15.05%) individuals who were eligible for participation and did participate.

**Fig. 2:**
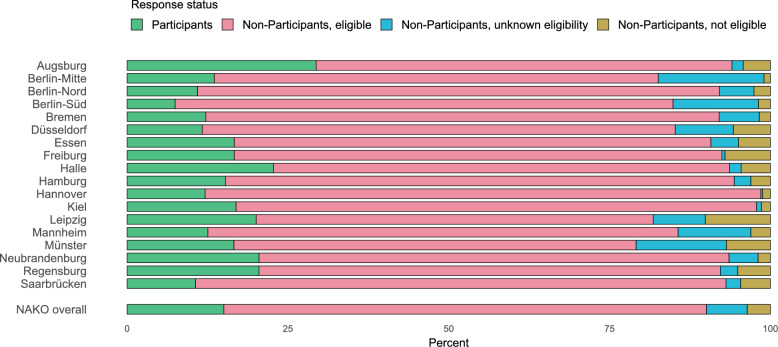
Percentage breakdown of response status categories across study centers. Note that the percentage of participants does not equal the response proportion, as the latter does not take into account non-eligible non-participants. Study centers are sorted alphabetically

The percentage breakdown of the four broad response categories (ineligible, unknown eligibility, eligible non-participants, participants) varied considerably across study centers (Fig. 2, Supplementary Table S1). Ineligible non-participants (NAKO overall 3.6%) were least common in Berlin-Mitte (1%) and most common in Leipzig (10.1%). Non-responder with unknown eligibility (NAKO overall 6.3%) were least common in Hannover (0.3%) and most common in Berlin-Mitte (16.4%). Eligible non-participants (NAKO overall 75.0%) were least common in Leipzig (61.7%) and most common in Hannover (86.4%).

### Overall response and differences across sex and age groups

**Fig. 3:**
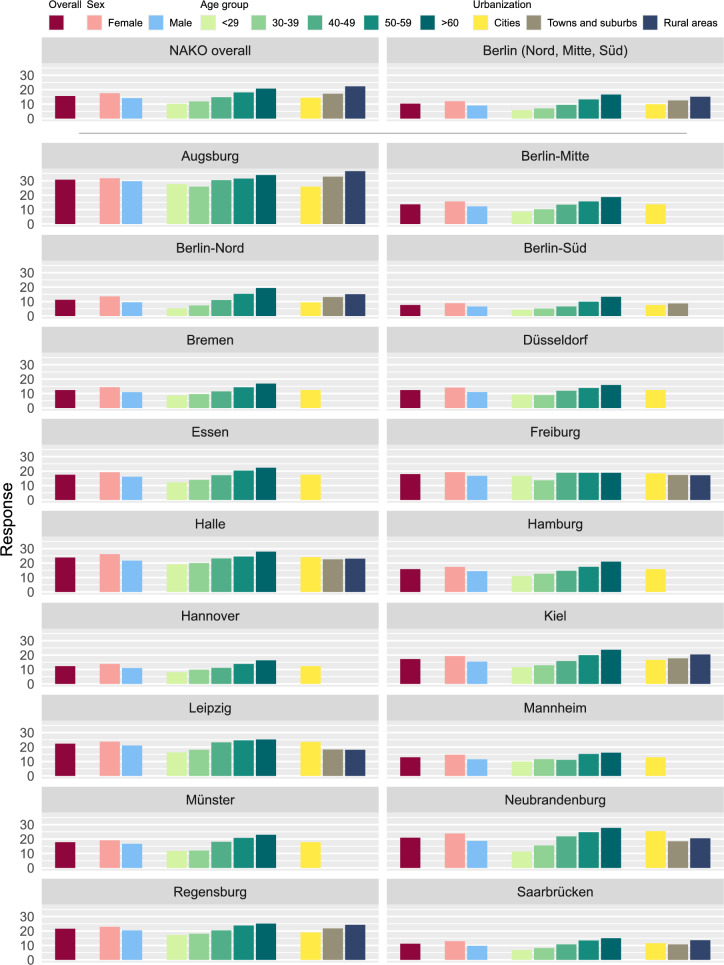
Response proportion (%) overall and stratified by sex, age group, and degree of urbanization for NAKO overall, the region of Berlin comprising 3 study centers, and all 18 individual study centers

The overall response across NAKO was 15.6%, but differed considerably across study centers, ranging from 30.7% in Augsburg down to 7.6% in Berlin-Süd (Table S2, Fig. 3). In all study centers response was higher among females (NAKO overall 17.5%) as compared to males (NAKO overall 14.1%) with a sex difference in overall response of 3.4 percentage points. Response was lowest in the youngest age group (< 29 years, NAKO overall: 10.2%) and increased up to 20.7% in the highest age group (> 60 years). A similar age gradient was observed in all study regions except Freiburg where no clear pattern was evident.

### Frequency of non-mandatory recruitment steps and response

Study centers varied in their use of non-mandatory recruitment steps (Supplementary Table S3). Eleven out of the 18 study centers used the option of sending a third reminder to persons who had not yet responded. The availability of phone numbers prior to the start of recruitment varied considerably across study centers. In four study centers no phone numbers were available, in nine study centers phone numbers were available for less than 10% of all persons in the random sample, in four study centers the percentage was between 12 and 27%, and in one center (Augsburg) the percentage of phone numbers was at 58%. The percentage of actual outbound phone call attempts prior to any active response by the invitees was slightly lower than the percentage of available phone numbers for 16 out of 18 study centers and was considerably lower for Augsburg and Neubrandenburg. Visual inspection of the relation between the use of non-mandatory recruitment steps in a study center and response did not reveal obvious dependencies (Fig. S1, Panel a). The number of reminder letters seemed not to result in differences in overall response (Fig. S1, Panel b), while fielding more outbound calls appeared to result in a higher response (Fig. S1, Panel c). Home visits were not routinely carried out in any of the study centers apart from pilot studies in two centers (Berlin-Mitte, Halle) [30].

### Degree of urbanization and response

**Fig. 4:**
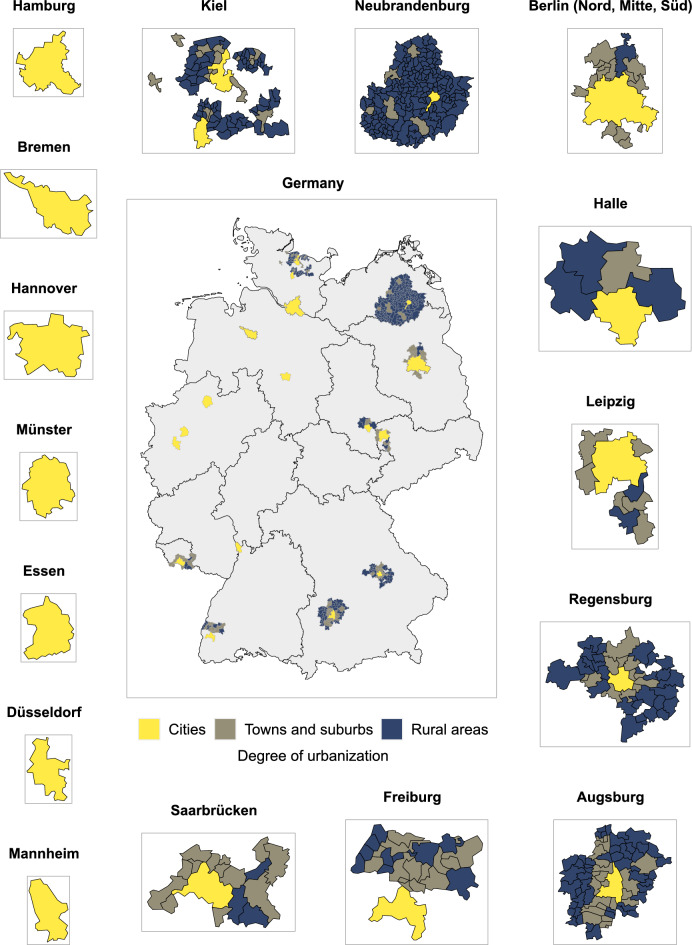
Degree of urbanization by study region and municipalities with NAKO participants. (administrative maps: © GeoBasis-DE / BKG 2018)

NAKO’s study base differed considerably among the study regions in terms of the degree of urbanization of their living areas (Fig. 4, Table 1, Supplementary Table S4). Overall, 80.2% of the study base lived in cities, 14.2% in towns and suburbs, and 5.7% in rural areas. In eight out of 18 study centers the entire study base lived in areas rated as cities (Berlin-Mitte, Bremen, Düsseldorf, Essen, Hamburg, Hannover, Mannheim, Münster) and in four study centers more than 80% of the study base lived in cities (Berlin-Nord, Berlin-Süd, Halle, Leipzig). In one study center more than 55% of the study base lived in towns and suburbs (Saarbrücken) and in one center more than 50% of the study base lived in rural areas (Neubrandenburg). In the remaining four study centers the study base was distributed more equally across urbanization categories (Augsburg, Freiburg, Kiel, Regensburg). In all study centers the percentage breakdown of urbanization categories for the eventual study sample did not differ notably from that of the respective study center’s study base. Note that individual comparisons for the three study centers in the study region Berlin were not possible because all jointly recruited in the city of Berlin and separate population data for their respective recruitment areas within the city were not available. Instead the study region of Berlin was analyzed and it did not show notable differences between the urbanization distribution in the study base, the invited sample, and the study sample.

**Fig. 5:**
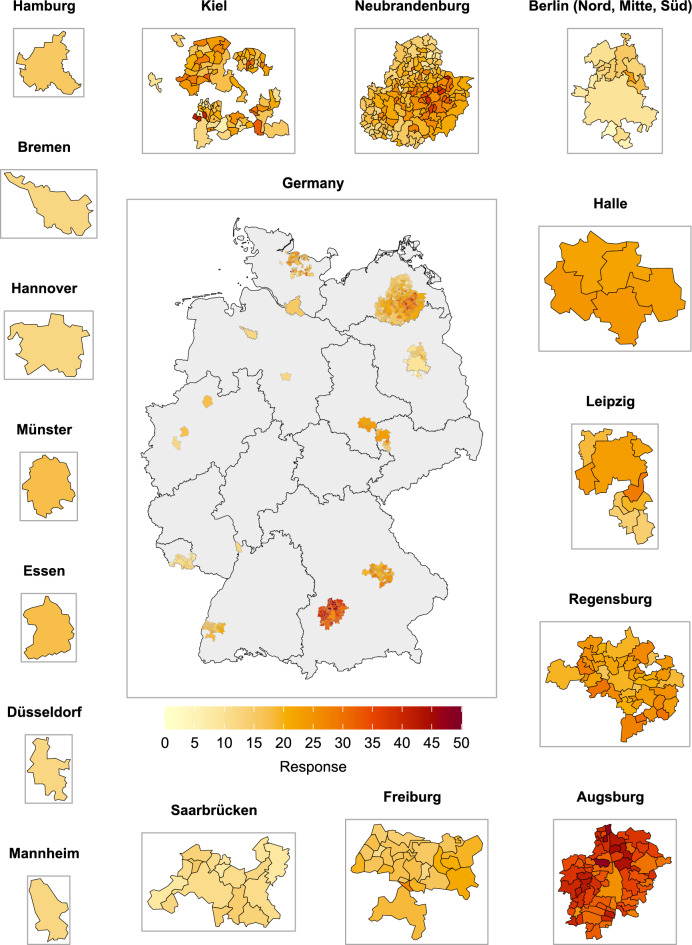
Response proportion (%) by study region and municipality. Municipalities with less than 15 invited persons (in the study region Kiel) are marked by black asterisks. (administrative maps: © GeoBasis-DE / BKG 2018)

Across all study regions response was highest in rural areas (22.3%), followed by towns and suburbs (17.2%), and lowest in cities (14.5%). In four out of the nine study regions that did not exclusively recruit from cities, the same response pattern was observed. In two study regions the response was highest in cities and in three study regions there was no clear pattern (Figs. 3 and 5, Supplementary Table S4).

### Survey weights and representativeness

**Fig. 6:**
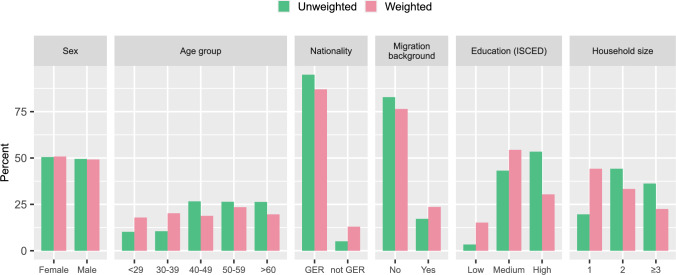
Comparision of the unweighted and weighted NAKO sample with respect to sex, age group, nationality, migration background, education, and household size

A comparison between the unweighted and the weighted sample (Fig. 6; see Supplementary Fig. S2 for individual centers) revealed that participants with non-German nationality and migration background were underrepresented in the NAKO sample overall as well as in the subsamples of almost all study regions. Only in the subsamples of Kiel and Regensburg the migration background closely mirrored that in the study base. In the overall NAKO sample as well as in all subsamples, participants with low and medium education were underrepresented, while highly educated participants were overrepresented. Participants from single households were underrepresented, while participants living in larger households (2 and ≥ 3 persons) were overrepresented in the overall NAKO sample and in the subsamples of all study centers. There was a notable discrepancy between the age distribution of the study sample and that of the study base, which, however, was intended by the sample design that aimed to oversample older age groups. Although the ratio between the sexes in the study sample was also determined by the sample design, it resembled the sex distribution in the study base.

## Discussion

NAKO is a large prospective multicenter cohort that examined more than 205,000 participants across Germany between 2014 and 2019, inviting more than 1.3 million individuals during the recruitment process. The resulting overall response proportion of 15.6% lies considerably below the 50% anticipated during the planning phase [18], but falls within the range reported by other large population-based cohort studies that conducted their baseline recruitment within the last two decades (e.g., UK-biobank 5.5% [31], Constances 7.3% [32], LifeLines 24.5% [33], China Kadoorie Biobank 30% [34], Japan Multi-institutional Collaborative Cohort 33.5% [35]). When compared to other large German population-based cohorts, the response in NAKO is considerably lower (e.g., EPIC 22.7—38.3% [36], KORA 65% [37], SHIP 69% [38]), but it is important to note that these cohorts recruited their baseline samples more than 20 years ago.

Although NAKO recruited according to a highly standardized protocol, response varied considerably across study centers. These differences could not be explained by differences in the use of additional reminder letters or phone calls alone. Study centers that sent out an additional third reminder letter to potential participants who had not yet responded did not seem to achieve higher response proportions compared to study centers that did not. Study centers that made more outbound calls to potential participants seemed to achieve slightly higher response proportions compared to study centers making less outbound calls, which would be consistent with previous reports [39]. It is important to note, however, that the use of telephone calls in this analysis was only quantified in terms of whether or not a potential participant was called. More detailed analyses that include the number of phone calls and their timing may provide additional insights [24]. Differences in overall response could also not be explained by differences in the degree of urbanization across study centers. However, despite differences in overall response, there were similar trends in the influence of participant characteristics on response across all study centers. Females were more likely to participate and the probability of participation increased with age. A comparison of the unweighted and the weighted NAKO study sample revealed that NAKO participants, as compared to the underlying population, were less likely to have a non-German nationality and a migration background, had higher education, and lived in larger households. These results are in line with previously reported trends (e.g., [1, 9]). It is important to note, however, that especially the differences in education and household size might at least partly be caused by the intended oversampling of older age groups, since both variables are associated with age. For instance, individuals in the lowest age group (< 29 years) are more likely to have not finished their education or to be living in smaller households. Furthermore, it cannot be ruled out that the observed differences in the percentage breakdown of nonresponse categories are caused by different decision-making rules by field staff of different study centers rather than by differences in the population under recruitment.

Overall, the lack of clear univariate explanations for response differences suggests that more complex multicausal mechanisms combining characteristics of potential participants, infrastructural differences across study regions, and differences in the recruitment efforts may be required to explain these results. Investigating the causes of nonresponse is not only of general interest for epidemiology [6], but particularly in cohort studies such as NAKO, because characteristics of recruitment at baseline may influence retention at follow-up stages [40–42]. NAKO has served as a resource for response experiments before [30, 41, 43–45] and in particular the extensive body of paradata collected with the MODYS software [24] offers future opportunities for nonresponse research.

NAKO provides survey weights that take into account the sampling design and the distribution of age, sex, nationality (German vs. non-German), migration status, education, and household size. Survey weights for the whole NAKO study sample as well as for the Level 2 and magnetic resonance imaging subsamples are made available to researchers along with the NAKO data. It is recommended that these weights be used whenever descriptive results (e.g., estimates of prevalence, risk, or exposure) from NAKO are generalized to the general population. Firstly, the NAKO sample differs from its source population already by design due to the intended age-sex distribution. Secondly, NAKO’s complex sampling design and the practice of drawing several successive random samples from the same source population very likely resulted in unequal inclusion probabilities, which are known to bias estimates [17, 46]. Finally, it is reasonable to assume that NAKO, like other large population-based cohort studies (e.g., [47]), was subject to self-selection effects during recruitment (e.g., “healthy volunteer bias”). For other analyses, there is no general recommendation and the use of weights should be decided on a case-by-case basis. For instance, while it is often not advisable to use correction weights when estimating complex models, because it may be difficult to satisfy very specific model assumptions (e.g., [48]), there are also exceptions to this rule (e.g., for causal modeling see [49]). It is crucial to note, however, that the intention of weighting was not to reach representativeness at the level of the German population as a whole, but representativeness at the level of each of the 16 study regions.

## Strengths and limitations

For a discussion of strengths and limitations of the NAKO cohort in general, the reader is referred to the cohort profile [18].

Since study regions were not randomly selected within Germany the recruited sample is unlikely to be representative of the whole German population, possibly limiting the generalizability of prevalence estimates for diseases and risk factors. Furthermore, the response proportion was low and the differences observed between the unweighted and the weighted NAKO study sample suggest that nonresponse was differential with respect to socioeconomic characteristics. For etiological research questions and prospective cohorts in particular, however, representativeness of the study sample is of less concern [11]. In addition, the generalizability of findings also depends on the particular endpoints of interest and should be assessed in each case separately.

Recruitment in NAKO was based on a highly standardized protocol that nevertheless allowed for study center-specific adaptions. The observed differences in response and the percentage distribution of nonresponse categories across study centers, however, may indicate that even these standardization efforts and the training of field staff could still be improved. This should be considered in future studies.

## Conclusion and outlook

NAKO recruited more than 205.000 participants between 2014 and 2019, inviting a total of 1.3 million residents aged 20–69 years from 16 German regions. Despite the highly standardized recruitment protocol NAKO achieved only a low response proportion, replicating comparable results in other recent large epidemiologic cohort studies. The patterns of nonresponse observed are consistent with those reported in other studies, e.g., older individuals and females were more likely to participate, as were those with higher education and those without a migration background. Response was also higher in rural areas than in urban areas. Survey weights that take these differences into account are available with the NAKO data.

For NAKO, the successful completion of the baseline examinations shifted the focus from recruiting to retaining participants, which comes with a whole new set of challenges. Motivating individuals to enroll and stay enrolled thereafter remains one of the main challenges for cohort studies [50]. Although new digital technologies offer exciting new opportunities to reduce barriers to enrollment and ease the burden of participation (e.g., [51]), efforts to increase participation must also consider the personal motivations of potential participants. The opportunity to learn more about one’s own health status, to receive personalized medical advice, to contribute to scientific progress, and the prospect of gaining insight into research practice are among the reasons for participating in health research [52–54]. To meet these expectations, cohort studies also need novel strategies for communicating with their participants, for offering them self-benefits, and for involving them in the research process (e.g., [55, 56]).

## Supplementary Information

Below is the link to the electronic supplementary material.

Supplementary file1 (DOCX 628 KB)

## Data Availability

Access to and use of NAKO data can be obtained via an electronic application portal (https://transfer.nako.de).

## Footnotes

1 All percentages in this paragraph refer to the total number of 1,364,918 individuals in the random sample.

## References

[1] Galea S, Tracy M. Participation rates in epidemiologic studies. Ann Epidemiol. 2007;17:643–53. 10.1016/j.annepidem.2007.03.013. 17553702 10.1016/j.annepidem.2007.03.013

[2] Groves RM. Nonresponse rates and nonresponse bias in household surveys. Public Opin Q. 2006;70(5):646–75.

[3] Morton LM, Cahill J, Hartge P. Reporting participation in epidemiologic studies: a survey of practice. Am J Epidemiol. 2006;163(3):197–203. 10.1093/aje/kwj036. 16339049 10.1093/aje/kwj036

[4] Nohr EA, Liew Z. How to investigate and adjust for selection bias in cohort studies. Acta Obstet Gynecol Scand. 2018;97(4):407–16. 10.1111/aogs.13319. 29415329 10.1111/aogs.13319

[5] Mindell JS, Giampaoli S, Goesswald A, et al. Sample selection, recruitment and participation rates in health examination surveys in Europe–experience from seven national surveys. BMC Med Res Methodol. 2015;15:78. 10.1186/s12874-015-0072-4. 26438235 10.1186/s12874-015-0072-4 PMC4595185

[6] Stang A. Nonresponse research - an underdeveloped field in epidemiology. Eur J Epidemiol. 2003;18:929–31. 14598921 10.1023/a:1025877501423

[7] van Zon SK, Scholtens S, Reijneveld SA, Smidt N, Bultmann U. Active recruitment and limited participant-load related to high participation in large population-based biobank studies. J Clin Epidemiol. 2016;78:52–62. 10.1016/j.jclinepi.2016.03.009. 27032874 10.1016/j.jclinepi.2016.03.009

[8] van Gelder M, Vlenterie R, IntHout J, Engelen L, Vrieling A, van de Belt TH. Most response-inducing strategies do not increase participation in observational studies: a systematic review and meta-analysis. J Clin Epidemiol. 2018;99:1–13. 10.1016/j.jclinepi.2018.02.019. 29518475 10.1016/j.jclinepi.2018.02.019

[9] Enzenbach C, Wicklein B, Wirkner K, Loeffler M. Evaluating selection bias in a population-based cohort study with low baseline participation: the LIFE-Adult-Study. BMC Med Res Methodol. 2019;19(1):135. 10.1186/s12874-019-0779-8. 31262266 10.1186/s12874-019-0779-8 PMC6604357

[10] Jöckel KH, Stang A. Cohort studies with low baseline response may not be generalisable to populations with different exposure distributions. Eur J Epidemiol. 2013;28(3):223–7. 10.1007/s10654-013-9782-2. 23456137 10.1007/s10654-013-9782-2

[11] Rothman KJ, Gallacher JE, Hatch EE. Why representativeness should be avoided. Int J Epidemiol. 2013;42(4):1012–4. 10.1093/ije/dys223. 24062287 10.1093/ije/dys223 PMC3888189

[12] Stang A, Jöckel KH. Studies with low Response Proportions may be less biased than Studies with high Response Proportions. Am J Epidemiol. 2004;159(2):204–10. 14718223 10.1093/aje/kwh009

[13] Groves RM, Couper MP, Presser S, et al. Experiments in producing nonresponse bias. Public Opin Q. 2006;70:720–36.

[14] Lacey JV Jr, Savage KE. 50% Response rates: half-empty, or half-full? Cancer Causes Control. 2016;27(6):805–8. 10.1007/s10552-016-0748-z. 27100357 10.1007/s10552-016-0748-z PMC9005206

[15] Nohr EA, Frydenberg M, Henriksen TB, Olsen J. Does low participation in cohort studies induce bias? Epidemiology. 2006;17(4):413–8. 10.1097/01.ede.0000220549.14177.60. 16755269 10.1097/01.ede.0000220549.14177.60

[16] Edwards PJ, Roberts I, Clarke MJ, et al. Methods to increase response to postal and electronic questionnaires. Cochrane Database Syst Rev. 2009;2010(3):MR000008. 10.1002/14651858.MR000008.pub4. 10.1002/14651858.MR000008.pub4 PMC8941848 19588449

[17] Sand M, Kunz T. Gewichtung in der praxis. Mannheim: GESIS–Leibniz-Institut für Sozialwissenschaften. 2020;10:4.

[18] Peters A, Peters A, et al. Framework and baseline examination of the german national cohort (NAKO). Eur J Epidemiol. 2022;37(10):1107–24. 10.1007/s10654-022-00890-5. 36260190 10.1007/s10654-022-00890-5 PMC9581448

[19] German National Cohort Consortium. The german national cohort: aims, study design and organization. Eur J Epidemiol. 2014;29(5):371–82. 10.1007/s10654-014-9890-7. 24840228 10.1007/s10654-014-9890-7 PMC4050302

[20] Schipf S, Schone G, Schmidt B, et al. The baseline assessment of the German National Cohort (NAKO Gesundheitsstudie): participation in the examination modules, quality assurance, and the use of secondary data. Bundesgesundheitsblatt Gesundheitsforschung Gesundheitsschutz. 2020;63(3):254–66. 10.1007/s00103-020-03093-z. 32047976 10.1007/s00103-020-03093-z

[21] Kuss O, Becher H, Wienke A, et al. Statistical analysis in the german national cohort (NAKO) - specific aspects and general recommendations. Eur J Epidemiol. 2022;37(4):429–36. 10.1007/s10654-022-00880-7. 35653006 10.1007/s10654-022-00880-7 PMC9187540

[22] The American Association for Public Opinion Research. Standard Definitions: Final Dispositions of Case Codes and Outcome Rates for Surveys. 10th ed: AAPOR; 2023.

[23] Dijkstra L, Florczyk AJ, Freire S, et al. Applying the Degree of Urbanisation to the globe: A new harmonised definition reveals a different picture of global urbanisation. J Urban Econ. 2021;125:103312. 10.1016/j.jue.2020.103312.

[24] Reineke A, Pigeot I, Ahrens W, Rach S. MODYS – a modular control and documentation system for epidemiological studies. In: Bammann K, Lissner L, Pigeot I, Ahrens W, editors. Instruments for health surveys in children and adolescents. Cham: Springer Nature Switzerland; 2018. p. 25–45.

[25] Kreuter F. Improving surveys with paradata : analytic uses of process information. Hoboken, New Jersey: Wiley & Sons; 2013.

[26] Horvitz DG, Thompson DJ. A generalization of sampling without replacement from a Finite Universe. J Am Stat Assoc. 1952;47(260):663–85. 10.1080/01621459.1952.10483446.

[27] Research Data Centres of the Federal Statistical Office and Statistical Offices of the Federal States of Germany. https://www.regionalstatistik.de/genesis/online/table/ [12411–02–03–5, 12211-Z-08, 12411–03–03–4-B, 12211-Z-05, 12211-Z-10], own calculations.

[28] van Buuren S, Groothuis-Oudshoorn K. Mice: multivariate imputation by chained equations in R. J Stat Softw. 2011;45(3):1–67. 10.18637/jss.v045.i03.

[29] Kolenikov S. Calibrating survey data using iterative proportional fitting (raking). Stand Genomic Sci. 2014;14(1):22–59. 10.1177/1536867x1401400104.

[30] Krist L, Bedir A, Fricke J, Kluttig A, Mikolajczyk R. The effect of home visits as an additional recruitment step on the composition of the final sample: a cross-sectional analysis in two study centers of the German National Cohort (NAKO). BMC Med Res Methodol. 2021;21(1):176. 10.1186/s12874-021-01357-z. 34425747 10.1186/s12874-021-01357-z PMC8383386

[31] Fry A, Littlejohns TJ, Sudlow C, et al. Comparison of sociodemographic and health-related characteristics of UK biobank participants with those of the general population. Am J Epidemiol. 2017;186(9):1026–34. 10.1093/aje/kwx246. 28641372 10.1093/aje/kwx246 PMC5860371

[32] Goldberg M, Carton M, Descatha A, et al. CONSTANCES: a general prospective population-based cohort for occupational and environmental epidemiology: cohort profile. Occup Environ Med. 2017;74(1):66–71. 10.1136/oemed-2016-103678. 27884936 10.1136/oemed-2016-103678 PMC5241503

[33] Sijtsma A, Rienks J, van der Harst P, Navis G, Rosmalen JGM, Dotinga A. Cohort Profile Update: Lifelines, a three-generation cohort study and biobank. Int J Epidemiol. 2022;51(5):e295–302. 10.1093/ije/dyab257. 34897450 10.1093/ije/dyab257 PMC9558073

[34] Chen Z, Chen J, Collins R, et al. China Kadoorie Biobank of 0.5 million people: survey methods, baseline characteristics and long-term follow-up. Int J Epidemiol. 2011;40(6):1652–66. 10.1093/ije/dyr120. 22158673 10.1093/ije/dyr120 PMC3235021

[35] Takeuchi K, Naito M, Kawai S, et al. Study profile of the japan multi-institutional collaborative cohort (J-MICC) study. J Epidemiol. 2021;31(12):660–8. 10.2188/jea.JE20200147. 32963210 10.2188/jea.JE20200147 PMC8593573

[36] Boeing H, Korfmann A, Bergmann MM. Recruitment procedures of EPIC-germany. Ann Nutr Metab. 1999;43(4):205–15. 10.1159/000012787. 10592369 10.1159/000012787

[37] Hoffmann W, Terschuren C, Holle R, et al. The problem of response in epidemiologic studies in Germany (Part II). Gesundheitswesen. 2004;66(8–9):482–91. 10.1055/s-2004-813094. 15372348 10.1055/s-2004-813094

[38] Latza U, Stang A, Bergmann M, et al. The problem of response in epidemiological studies in Germany (part I). Gesundheitswesen. 2004;66(5):326–36. 10.1055/s-2004-813093. 15141353 10.1055/s-2004-813093

[39] Stang A, Moebus S, Dragano N, et al. Baseline recruitment and analyses of nonresponse of the Heinz nixdorf recall study: identifiability of phone numbers as the major determinant of response. Eur J Epidemiol. 2005;20(6):489–96. 10.1007/s10654-005-5529-z. 16121757 10.1007/s10654-005-5529-z

[40] Langeheine M, Pohlabeln H, Ahrens W, Rach S. Consequences of an extended recruitment on participation in the follow-up of a child study: results from the German IDEFICS cohort. Paediatr Perinat Epidemiol. 2017;31(1):76–86. 10.1111/ppe.12328. 27873339 10.1111/ppe.12328

[41] Rach S, Gunther K, Hadeler B. Participants who were difficult to recruit at baseline are less likely to complete a follow-up questionnaire - results from the German National Cohort. BMC Med Res Methodol. 2020;20(1):187. 10.1186/s12874-020-01073-0. 32646374 10.1186/s12874-020-01073-0 PMC7346423

[42] Teague S, Youssef GJ, Macdonald JA, et al. Retention strategies in longitudinal cohort studies: a systematic review and meta-analysis. BMC Med Res Methodol. 2018;18(1):151. 10.1186/s12874-018-0586-7. 30477443 10.1186/s12874-018-0586-7 PMC6258319

[43] Langeheine M, Pohlabeln H, Ahrens W, Gunther K, Rach S. Study invitations with envelopes made from recycled paper do not increase likelihood of active responses or study participation in the German National Cohort. BMC Res Notes. 2019;12(1):468. 10.1186/s13104-019-4510-y. 31366371 10.1186/s13104-019-4510-y PMC6670214

[44] Winkler V, Leitzmann M, Obi N, et al. Response in individuals with and without foreign background and application to the National Cohort in Germany: which factors have an effect? Int J Public Health. 2014;59(3):555–63. 10.1007/s00038-013-0539-1. 24390621 10.1007/s00038-013-0539-1

[45] Reiss K, Dragano N, Ellert U, et al. Comparing sampling strategies to recruit migrants for an epidemiological study. Results from a German feasibility study. Eur J Public Health. 2014;24(5):721–6. 10.1093/eurpub/cku046. 24872519 10.1093/eurpub/cku046

[46] Sand M, Bruch C, Felderer B, Schaurer I, Kolb J-P, Weyandt K. Creating Design Weights for a Panel Survey with Multiple Refreshment Samples: A General Discussion with an Application to a Probability-Based Mixed-Mode Panel. methods, data, analyses (in press).

[47] van Alten S, Domingue BW, Faul J, Galama T, Marees AT. Reweighting UK Biobank corrects for pervasive selection bias due to volunteering. Int J Epidemiol. 2024. 10.1093/ije/dyae054. 38715336 10.1093/ije/dyae054 PMC11076923

[48] Gelman A. Struggles with survey weighting and regression modeling. Stat Sci. 2007. 10.1214/088342306000000691.

[49] Hernan MA, Robins JM. Estimating causal effects from epidemiological data. J Epidemiol Community Health. 2006;60(7):578–86. 10.1136/jech.2004.029496. 16790829 10.1136/jech.2004.029496 PMC2652882

[50] Murray AL, Xie T. Engaging adolescents in contemporary longitudinal health research: strategies for promoting participation and retention. J Adolesc Health. 2024;74(1):9–17. 10.1016/j.jadohealth.2023.06.032. 37690009 10.1016/j.jadohealth.2023.06.032

[51] Ortmann J, Heise JK, Janzen I, et al. Suitability and user acceptance of the eResearch system “Prospective Monitoring and Management App (PIA)”-The example of an epidemiological study on infectious diseases. PLoS ONE. 2023;18(1):e0279969. 10.1371/journal.pone.0279969. 36595548 10.1371/journal.pone.0279969 PMC9810156

[52] Merz S, Jaehn P, Pischon T, et al. Investigating people’s attitudes towards participating in longitudinal health research: an intersectionality-informed perspective. Int J Equity Health. 2023;22(1):23. 10.1186/s12939-022-01807-0. 36721141 10.1186/s12939-022-01807-0 PMC9887766

[53] Nobile H, Bergmann MM, Moldenhauer J, Borry P. Participants’ accounts on their decision to join a cohort study with an attached biobank: a qualitative content analysis study within two german studies. J Empir Res Hum Res Ethics. 2016;11(3):237–49. 10.1177/1556264616657463. 27381010 10.1177/1556264616657463

[54] Nobile H, Borry P, Pischon T, et al. Participants’ decision to enroll in cohort studies with biobanks: quantitative insights from two German studies. Per Med. 2017;14(6):477–85. 10.2217/pme-2017-0049. 29749857 10.2217/pme-2017-0049

[55] Ruckert-Eheberg IM, Heier M, Simon M, Kraus M, Peters A, Linkohr B. Public attitudes towards personal health data sharing in long-term epidemiological research: a Citizen Science approach in the KORA study. BMC Public Health. 2024;24(1):2317. 10.1186/s12889-024-19730-0. 39187842 10.1186/s12889-024-19730-0 PMC11348671

[56] Herrera-Espejel PS, Rach S. The use of machine translation for outreach and health communication in epidemiology and public health: scoping review. JMIR Public Health Surveill. 2023;9:e50814. 10.2196/50814. 37983078 10.2196/50814 PMC10696499

