# Supplementary material for: The baseline examinations of the German National Cohort (NAKO): recruitment protocol, response, and weighting
Source: Eur J Epidemiol. 2025 Apr 22;40(4):475–89. doi: 10.1007/s10654-025-01219-8 (PMC12145326; doi:10.1007/s10654-025-01219-8)

**Supplementary Information**

**The baseline examinations of the German National Cohort (NAKO): recruitment protocol, response, and weighting**

Stefan Rach^1,*^, Matthias Sand^2^, Achim Reineke^3^, Heiko Becher^4^, Karin Halina Greiser^5^, Kathrin Wolf^6^, Kerstin Wirkner^7^, Carsten Oliver Schmidt^8^, Sabine Schipf^8^, Karl-Heinz Jöckel^9,1^, Lilian Krist^10^, Wolfgang Ahrens^1^, Hermann Brenner^11^, Stefanie Castell^12^, Sylvia Gastell^13^, Volker Harth^14^, Bernd Holleczek^15^, Till Ittermann^8^, Stefan Janisch-Fabian^3^, André Karch^16^, Thomas Keil^10,17,18^, Carolina J. Klett-Tammen^12^, Alexander Kluttig^19,20^, Oliver Kuß^21^, Michael  Leitzmann^22^, Wolfgang Lieb^23^, Claudia Meinke-Franze^8^, Karin B. Michels^24^, Rafael Mikolajczyk^25,20^, Ilais Moreno Velásquez^26^, Nadia Obi^14^, Cara Övermöhle^23^, Annette Peters^6,27^, Tobias Pischon^26,28,29^, Susanne Rospleszcz^30,6^, Börge Schmidt^31^, Matthias B. Schulze^13,32^, Andreas Stang^31^, Henning Teismann^16^, Christine  Töpfer ^22^, Robert Wolff^33^, Kathrin Günther^1^

^1^ Leibniz Institute for Prevention Research and Epidemiology - BIPS, Department of Epidemiological Methods and Etiological Research, Bremen, Germany.

^2^ GESIS—Leibniz Institute for the Social Sciences, Department of Survey Design and Methodology, Mannheim, Germany.

^3^ Leibniz Institute for Prevention Research and Epidemiology - BIPS, Department of Biometry and Data Management, Bremen, Germany.

^4^ Institute of Global Health, University Hospital Heidelberg, Heidelberg, Germany.

^5^ Division of Cancer Epidemiology, German Cancer Research Center (DKFZ), Heidelberg, Germany.

^6^ Institute of Epidemiology, Helmholtz Zentrum München - German Research Center for Environmental Health (GmbH), Neuherberg, Germany.

^7^ Leipzig Research Centre for Civilization Diseases, University of Leipzig, Leipzig, Germany.

^8^ Institute for Community Medicine, University Medicine Greifswald, Greifswald, Germany.

^9^ Medical Faculty, University of Duisburg-Essen, University Hospital Essen, Essen, Germany.

^10^ Institute of Social Medicine, Epidemiology and Health Economics, Charité - Universitätsmedizin Berlin, Berlin, Germany.

^11^ Division of Clinical Epidemiology and Aging Research, German Cancer Research Center (DKFZ), Heidelberg, Germany.

^12^ Department of Epidemiology, Helmholtz Centre for Infection Research (HZI), Braunschweig, Germany.

^13^ German Institute of Human Nutrition Potsdam-Rehbruecke, Nuthetal, Germany.

^14^ Institute for Occupational and Maritime Medicine (ZfAM), University Medical Center Hamburg-Eppendorf, Hamburg, Germany.

^15^ Saarland Cancer Registry, Saarbrücken, Germany.

^16^ Institute of Epidemiology and Social Medicine, Münster, Germany.

^17^ Institute of Clinical Epidemiology and Biometry, University of Würzburg, Würzburg, Germany.

^18^ State Institute of Health I, Bavarian Health and Food Safety Authority, Erlangen, Germany.

^19^ Martin-Luther University Halle-Wittenberg, Institute of Medical Epidemiology, Biostatistics and Informatics, Halle (Saale), Germany.

^20^ Interdisciplinary Center for Health Sciences, Medical Faculty of the Martin-Luther-University Halle-Wittenberg, Halle (Saale), Germany.

^21^ German Diabetes Center (DDZ), Institute for Biometrics and Epidemiology, Düsseldorf, Germany.

^22^ Department of Epidemiology and Preventive Medicine, University of Regensburg, Regensburg, Germany.

^23^ Institute of Epidemiology, Kiel University, Kiel, Germany.

^24^ Institute for Prevention and Cancer Epidemiology, Faculty of Medicine and Medical Center, University of Freiburg, Freiburg, Germany.

^25^ Institute of Medical Epidemiology, Biostatistics, and Informatics, Medical Faculty of the Martin-Luther-University Halle-Wittenberg, Halle (Saale), Germany.

^26^ Max-Delbrück-Center for Molecular Medicine in the Helmholtz Association (MDC), Molecular Epidemiology Research Group, Berlin, Germany.

^27^ Chair of Epidemiology, Institute for Medical Information Processing, Biometry and Epidemiology, Medical Faculty, Ludwig-Maximilians-Universität München, Munich, Germany.

^28^ Max-Delbrück-Center for Molecular Medicine in the Helmholtz Association (MDC), Biobank Technology Platform, Berlin, Germany.

^29^ Charité - Universitätsmedizin Berlin, corporate member of Freie Universität Berlin and Humboldt-Universität zu Berlin, Berlin, Germany.

^30^ Department of Diagnostic and Interventional Radiology, University Medical Center Freiburg, Faculty of Medicine, University of Freiburg, Freiburg, Germany.

^31^ Institute for Medical Informatics, Biometry and Epidemiology, University Hospital Essen, University Duisburg-Essen, Essen, Germany.

^32^ Institute of Nutritional Science, University of Potsdam, Nuthetal, Germany.

^33^ Trusted Third Party of the University Medicine Greifswald, Greifswald, Germany.

***Correspondence to:**

Dr. Stefan Rach

Leibniz Institute for Prevention Research and Epidemiology – BIPS

Department of Epidemiological Methods and Etiological Research

Achterstr. 30, 28359 Bremen, Germany

rach@leibniz-bips.de, sec-epi@leibniz-bips.de

**Contents**

**Supplementary Methods**

**Methods M1:** Calculation of survey weights

**Supplementary Tables**

**Supplementary Table S1** Frequency of non-response categories and response

**Supplementary Table S2** Response proportion (%) by sex and age group for all study centers / study regions.

**Supplementary Table S3** Frequency of non-mandatory recruitment steps and overall response in study centers

**Supplementary Table S4** Composition of study base (residents 20-69 years), invited sample, and study sample with respect to the degree of urbanization (DEGURBA) of the residential area and response stratified by study region and DEGURBA

**Supplementary Figures**

**Supplementary Figure S1** Response proportion (%) and frequency of non-mandatory recruitment steps.

**Supplementary Figure S2** Comparision of the unweighted and weighted samples for 16 NAKO study regions with respect to sex, age group, nationality, migration background, education, and household size

**Supplementary methods M1: Calculation of survey weights**

Survey weights are available for the whole sample as well as for the subsample completing the in-depth examinations (Level 2 program) and the subsample completing magnetic resonance imaging (MRI). The survey weights are made available to researchers along with the NAKO data via an electronic application portal (https://transfer.nako.de).

**Survey weights for the full NAKO sample**

Survey weights for the full NAKO sample were determined in a two-step procedure consisting of design weighting and calibration (1). Design weights take into account the sample design and correct for unequal inclusion probabilities while calibration weights adjust for differential nonresponse and underrepresentation.

*Design weights*

Since the NAKO study sample had an intended age distribution that differed from the age distribution of the source population in its study regions and the study regions differed regarding their population density and infrastructural characteristics (i.e., in the degree of urbanization of their municipalities), the sample design may have resulted in unequal inclusion probabilities in some regions. Moreover, since the sampling had been conducted over several years, the probability of being randomly selected for individuals might be unequal depending on their age (similar to panel studies with refreshments (2)).

Design weights were therefore calculated to correct for unequal inclusion probabilities of individual participants of the study. Official population data from the intercensal population updates provided by the Federal Statistical Office (3) for the years 2014 to 2019 at the level of municipalities (“Gemeinde”) were aggregated to match the NAKO age groups (20-39, 40-49, 50-59, 60+). For each municipality covered by the study regions, individual inclusion probabilities were calculated in all five age groups for both sexes separately. The inclusion probability only takes into account the probability of an individual being randomly selected, but not whether a selected individual enrolled in the study or which reasons led to not enrolling. Design weights were calculated using the Horvitz-Thompson-Estimator (4) by taking the inverse of the inclusion probability. Only the design weights of participants were subjected to the calibration step of the weighting procedure. The design weights of non-participants were discarded and not used in further steps of the weighting procedure.

*Calibration weights*

In a second step calibration weights were calculated to account for differential nonresponse and to reduce the bias and variance of the estimated parameters. Variables used for calibration were age-group, sex, nationality (German vs. non-German), education (low: ISCED97 1-2, vs. medium: ISCED97 3-4 vs. high: ISCED97 5-6), migration background (yes vs. no), and household size (1 vs. 2 vs. ≥3 persons). Missing values in the calibration variables were imputed using a fully chained equation modelling approach implemented in R’s MICE Package (5). Data from the official German Microzensus (3) were used to determine marginal distributions of the calibration variables in the general population aged between 20-69 years for each administrative district (“Kreis”) covered by the study regions. Using these marginal distributions, calibration weights of the design weighted estimator were calculated by iterative proportional fitting (“raking”) (6) separately for each administrative district.

*Total survey weights*

Survey weights were obtained by multiplying design and calibration weights. Survey weights were trimmed to the 1st and 99th percentile to lower the variance of the weights and reduce the influence of outliers. Finally, for each study center separately, the weights were rescaled (normalized) so that their sum equaled the sample size of the respective study center. Two sets of weights are available: one with the samples of the three study centers in the study region Berlin (Berlin-Mitte, Berlin-Nord and Berlin-Süd) were joined before rescaling (variable wgt_total_**16**sc in the NAKO data set) and one with the three centers were rescaled individually (variable wgt_total_**18**sc in the NAKO data set). For analyses of the full NAKO sample it is recommended to use the first set of weights (wgt_total_16sc). The second set should only be used when data from any of the three Berlin study centers are analyzed separately.

**Survey weights for subsamples (Level 2 program and MRI)**

Survey weights are available also for the subsamples completing the in-depth examinations (Level 2 program) and the subsample completing MRI. Although participants were randomly selected for inclusion in the Level 2 and/or MRI subsamples, they were free to decline and only complete the Level 1 program instead. Since it cannot be ruled out that these self-selection processes caused systematic differences between the subgroup and the full sample, survey weights for the subgroups were determined for each subgroup separately in a three-step procedure consisting of design weighting, propensity score weighting, and calibration. Propensity score weights account for differences between treatment and comparison groups caused by group (self-)assignment.

*Design weights*

The design weights calculated for the full NAKO sample were used for the weighting of subsamples. Only weights of the respective subgroup were used.

*Propensity score weighting*

To account for selection differences between a subgroup that completed additional examinations (Level 2 program or MRI) and the group that did not, propensity score weights were calculated. Logistic generalized linear models were fitted for each sex separately using the group assignment as outcome and sociodemographic characteristics (age-group, nationality, education, migration background, household size) and health related variables (e.g., information on preexisting conditions, frequency of visits to physicians and specialists, history of smoking and alcohol use) as regressors. Propensity score weights were calculated by taking the inverse of the propensity of being included in the subgroup as predicted by the regression model. Only propensity score weights for participants included in the respective subgroups were subjected to the calibration step of the weighting procedure.

*Calibration weights*

Calibration weights for subgroups were determined according to the same procedure as described for the full sample.

*Total survey weights*

Survey weights for subgroups were calculated by multiplying design weights, propensity score weights, and calibration weights, and trimmed to the 1st and 99th percentile afterwards. Finally, for each study center separately, the weights were rescaled (normalized) so that their sum equaled the sample size of the subgroup in the respective study center using the same procedure as for the full NAKO sample.

*References*

1. Sand M, Kunz T. Gewichtung in der Praxis. Mannheim: GESIS – Leibniz-Institut für Sozialwissenschaften 2020.

2. Sand M, Bruch C, Felderer B, Schaurer I, Kolb J-P, Weyandt K. Creating Design Weights for a Panel Survey with Multiple Refreshment Samples: A General Discussion with an Application to a Probability-Based Mixed-Mode Panel. methods, data, analyses (in press).

3. Research Data Centres of the Federal Statistical Office and Statistical Offices of the Federal States of Germany. <https://www.regionalstatistik.de/genesis/online/table/> [12411-02-03-5, 12211-Z-08, 12411-03-03-4-B, 12211-Z-05, 12211-Z-10], own calculations.

4. Horvitz DG, Thompson DJ. A Generalization of Sampling Without Replacement from a Finite Universe. Journal of the American Statistical Association. 1952;47(260):663-85. doi:10.1080/01621459.1952.10483446

5. van Buuren S, Groothuis-Oudshoorn K. mice: Multivariate Imputation by Chained Equations in R. Journal of Statistical Software. 2011;45(3):1 - 67. doi:10.18637/jss.v045.i03

6. Kolenikov S. Calibrating Survey Data using Iterative Proportional Fitting (Raking). The Stata Journal. 2014;14(1):22-59. doi:10.1177/1536867x1401400104

**Supplementary Tables**

**Table S1** Frequency of non-response categories and response

| Study center | Non-participants | | | Participants | Response |
| --- | --- | --- | --- | --- | --- |
|  | Not eligible | Unknown eligibility | Eligible |  |  |
|  | n (%) | n (%) | n (%) | n (%) | % |
| Augsburg | 2,962 (4.2) | 1,224 (1.7) | 45,381 (64.6) | 20,628 (29.4) | 30.7 |
| Berlin-Mitte | 812 (1.0) | 13,397 (16.4) | 56,276 (69.0) | 11,055 (13.6) | 13.7 |
| Berlin-Nord | 2,349 (2.5) | 4,956 (5.4) | 74,890 (81.1) | 10,114 (11.0) | 11.2 |
| Berlin-Süd | 2,498 (1.9) | 17,858 (13.3) | 104,106 (77.4) | 10,045 (7.5) | 7.6 |
| Bremen | 1,479 (1.7) | 5,361 (6.2) | 68,455 (79.8) | 10,492 (12.2) | 12.4 |
| Düsseldorf | 4,507 (5.7) | 7,098 (9.0) | 57,726 (73.5) | 9,161 (11.7) | 12.4 |
| Essen | 3,177 (5.0) | 2,732 (4.3) | 47,419 (74.1) | 10,660 (16.7) | 17.5 |
| Freiburg | 4,284 (7.1) | 292 (0.5) | 46,030 (75.8) | 10,110 (16.7) | 17.9 |
| Halle | 2,032 (4.6) | 796 (1.8) | 31,656 (70.9) | 10,143 (22.7) | 23.8 |
| Hamburg | 2,002 (3.0) | 1,719 (2.6) | 52,381 (79.1) | 10,111 (15.3) | 15.7 |
| Hannover | 1 009 (1.2) | 239 (0.3) | 71,767 (86.4) | 10,066 (12.1) | 12.3 |
| Kiel | 781 (1.4) | 422 (0.8) | 45,414 (80.9) | 9,511 (16.9) | 17.2 |
| Leipzig | 5,477 (10.1) | 4,399 (8.1) | 33,445 (61.7) | 10,876 (20.1) | 22.3 |
| Mannheim | 2,480 (3.0) | 9,283 (11.3) | 59,996 (73.1) | 10,301 (12.6) | 12.9 |
| Münster | 4,127 (6.8) | 8,484 (14.0) | 37,788 (62.5) | 10,039 (16.6) | 17.8 |
| Neubrandenburg | 2 041 (1.9) | 4,863 (4.5) | 78,429 (73.1) | 22,024 (20.5) | 20.9 |
| Regensburg | 2 475 (5.1) | 1,331 (2.7) | 35,102 (71.7) | 10,033 (20.5) | 21.6 |
| Saarbrücken | 4 371 (4.6) | 2,140 (2.3) | 77,786 (82.5) | 10 045 (10.6) | 11.2 |
| Total | 48,863 (3.6) | 86,594 (6.3) | 1,024,047 (75.0) | 205 414 (15.0) | 15.6 |

**Table S2** Response proportion (%) by sex and age group for all study centers / study regions.

| **0** | **Sex** | **Age group** | | | | | **All ages** |
| --- | --- | --- | --- | --- | --- | --- | --- |
|  |  | **<29** | **30-39** | **40-49** | **50-59** | **>60** |  |
| Augsburg | Female | 29.7 | 26.8 | 32.4 | 33.6 | 32.4 | 31.7 |
|  | Male | 25.8 | 25.0 | 28.6 | 29.7 | 35.4 | 29.7 |
|  | All | 27.6 | 25.9 | 30.4 | 31.5 | 33.9 | 30.7 |
| Berlin (Nord, Mitte, Süd) | Female | 6.8 | 8.3 | 11.3 | 15.5 | 17.6 | 12.0 |
|  | Male | 4.8 | 6.1 | 8.3 | 11.4 | 15.7 | 9.0 |
|  | All | 5.7 | 7.0 | 9.5 | 13.2 | 16.6 | 10.3 |
| Berlin-Mitte | Female | 10.1 | 11.9 | 15.7 | 18.6 | 19.5 | 15.6 |
|  | Male | 7.5 | 8.9 | 11.8 | 13.6 | 18.0 | 12.2 |
|  | All | 8.6 | 10.2 | 13.4 | 15.6 | 18.7 | 13.6 |
| Berlin-Nord | Female | 6.7 | 8.9 | 13.9 | 17.9 | 20.6 | 13.6 |
|  | Male | 4.3 | 6.1 | 9.0 | 13.3 | 18.2 | 9.5 |
|  | All | 5.3 | 7.3 | 11.0 | 15.3 | 19.4 | 11.2 |
| Berlin-Süd | Female | 5.0 | 5.9 | 7.5 | 11.9 | 14.3 | 8.9 |
|  | Male | 3.8 | 4.5 | 5.8 | 8.3 | 12.2 | 6.6 |
|  | All | 4.3 | 5.1 | 6.6 | 9.9 | 13.3 | 7.6 |
| Bremen | Female | 10.3 | 11.3 | 14.0 | 17.0 | 17.4 | 14.4 |
|  | Male | 7.3 | 8.1 | 9.7 | 12.4 | 16.3 | 10.9 |
|  | All | 8.6 | 9.6 | 11.5 | 14.3 | 16.9 | 12.4 |
| Düsseldorf | Female | 10.9 | 10.0 | 14.3 | 16.0 | 16.3 | 14.1 |
|  | Male | 8.0 | 8.1 | 10.1 | 12.1 | 15.5 | 11.0 |
|  | All | 9.3 | 8.9 | 11.9 | 13.8 | 15.9 | 12.4 |
| Essen | Female | 14.4 | 15.2 | 19.1 | 23.1 | 21.7 | 19.2 |
|  | Male | 10.3 | 12.7 | 15.6 | 18.1 | 22.9 | 16.1 |
|  | All | 12.2 | 13.9 | 17.1 | 20.3 | 22.3 | 17.5 |
| Freiburg | Female | 19.5 | 15.2 | 21.1 | 20.7 | 18.4 | 19.3 |
|  | Male | 14.6 | 12.3 | 17.1 | 17.3 | 19.3 | 16.7 |
|  | All | 16.7 | 13.6 | 18.8 | 18.8 | 18.8 | 17.9 |
| Halle | Female | 22.8 | 23.5 | 26.7 | 26.7 | 27.8 | 26.2 |
|  | Male | 16.3 | 17.1 | 20.2 | 22.5 | 28.0 | 21.7 |
|  | All | 19.2 | 20.0 | 23.2 | 24.5 | 27.9 | 23.8 |
| Hamburg | Female | 12.3 | 13.8 | 17.3 | 19.2 | 20.7 | 17.3 |
|  | Male | 10.0 | 11.6 | 12.6 | 15.9 | 21.4 | 14.4 |
|  | All | 11.0 | 12.6 | 14.7 | 17.4 | 21.0 | 15.8 |
| Hannover | Female | 10.1 | 11.1 | 13.1 | 15.4 | 16.4 | 13.8 |
|  | Male | 6.8 | 8.8 | 9.6 | 12.4 | 16.2 | 11.0 |
|  | All | 8.1 | 9.9 | 11.1 | 13.8 | 16.3 | 12.3 |
| Kiel | Female | 14.6 | 15.0 | 18.9 | 22.2 | 22.8 | 19.3 |
|  | Male | 9.2 | 11.0 | 13.6 | 17.9 | 24.6 | 15.4 |
|  | All | 11.6 | 12.9 | 15.8 | 19.9 | 23.7 | 17.2 |
| Leipzig | Female | 18.4 | 19.1 | 26.5 | 25.7 | 24.6 | 23.7 |
|  | Male | 14.7 | 17.2 | 20.8 | 23.5 | 25.8 | 21.1 |
|  | All | 16.3 | 18.1 | 23.2 | 24.6 | 25.2 | 22.3 |
| Mannheim | Female | 13.0 | 12.7 | 13.0 | 17.6 | 16.0 | 14.7 |
|  | Male | 8.2 | 10.8 | 9.8 | 13.2 | 16.1 | 11.5 |
|  | All | 10.0 | 11.6 | 11.1 | 15.2 | 16.1 | 12.9 |
| Münster | Female | 13.0 | 13.5 | 19.6 | 23.0 | 22.0 | 19.1 |
|  | Male | 10.7 | 10.8 | 16.8 | 19.0 | 24.0 | 16.7 |
|  | All | 11.7 | 12.0 | 18.1 | 20.8 | 22.9 | 17.8 |
| Neubrandenburg | Female | 13.8 | 18.9 | 25.5 | 27.7 | 28.3 | 23.8 |
|  | Male | 9.4 | 13.3 | 19.0 | 22.4 | 27.2 | 18.7 |
|  | All | 11.3 | 15.5 | 21.8 | 24.7 | 27.7 | 20.9 |
| Regensburg | Female | 19.0 | 20.4 | 22.9 | 25.5 | 23.4 | 22.9 |
|  | Male | 15.8 | 16.2 | 18.5 | 22.2 | 27.0 | 20.4 |
|  | All | 17.2 | 18.1 | 20.4 | 23.8 | 25.1 | 21.6 |
| Saarbrücken | Female | 8.0 | 9.8 | 12.7 | 15.4 | 15.5 | 12.9 |
|  | Male | 5.9 | 7.2 | 9.1 | 11.6 | 14.5 | 9.7 |
|  | All | 6.8 | 8.2 | 10.7 | 13.4 | 15.0 | 11.2 |
| NAKO overall | Female | 12.2 | 13.5 | 17.2 | 20.4 | 20.7 | 17.5 |
|  | Male | 8.8 | 10.4 | 13.0 | 16.2 | 20.7 | 14.1 |
|  | All | 10.2 | 11.8 | 14.8 | 18.1 | 20.7 | 15.6 |

**Table S3** Frequency of non-mandatory recruitment steps and overall response in study centers

| Study center | Reminder letters^a^ | Phone number at start of recruitment^b^ (%) | Outbound call attempts^c^ (%) | Response^d^ (%) |
| --- | --- | --- | --- | --- |
| Augsburg | 3 | 58 | 46 | 30.7 |
| Berlin-Mitte | 3 | 6 | 1 | 13.6 |
| Berlin-Nord | 2 | - | - | 11.2 |
| Berlin-Süd | 3 | - | 1 | 7.6 |
| Bremen | 3 | 17 | 14 | 12.4 |
| Düsseldorf | 2 | 2 | 2 | 12.4 |
| Essen | 3 | 8 | 8 | 17.5 |
| Freiburg | 3 | 6 | 5 | 17.9 |
| Halle | 3 | 18 | 16 | 23.8 |
| Hamburg | 3 | 3 | 3 | 15.8 |
| Hannover | 3 | 12 | 12 | 12.3 |
| Kiel | 2 | 5 | 3 | 17.2 |
| Leipzig | 2 | - | 3 | 22.3 |
| Mannheim | 3 | 7 | 6 | 12.9 |
| Münster | 2 | - | 3 | 17.8 |
| Neubrandenburg | 3 | 27 | 16 | 20.9 |
| Regensburg | 2 | 9 | 7 | 21.6 |
| Saarbrücken | 2 | 4 | 3 | 11.2 |

^a^ Number of reminder letters routinely sent out to non-respondents.

^b^ Percentage of persons with available phone numbers at the start of recruitment.

^c^ Percentage of persons for whom outbound calls were documented prior to an active response from the invited individual (third column). Note that the percentage of outbound calls may exceed the percentage of available phone numbers if phone numbers became available later in the recruitment process.

^d^ Overall response proportion in the study center.

**Table S4** Composition of study base (residents 20-69 years), invited sample, and study sample with respect to the degree of urbanization (DEGURBA) of the residential area and response stratified by study region and DEGURBA

| Study center / study region | Urbanization (DEGURBA) | Study base | |  | Invited sample | |  | Study sample | |  | Response (%) | |
| --- | --- | --- | --- | --- | --- | --- | --- | --- | --- | --- | --- | --- |
|  |  | n | % |  | n | % |  | n | % |  |  | |
| Augsburg | Cities | 190,434 | 42.0 |  | 27,927 | 41.5 |  | 7,242 | 35.1 |  | 25.9 | |
|  | Towns and suburbs | 173,143 | 38.2 |  | 26,654 | 39.6 |  | 8,754 | 42.4 |  | 32.8 | |
|  | Rural areas | 89,435 | 19.7 |  | 12,663 | 18.8 |  | 4,631 | 22.5 |  | 36.6 | |
|  | Total | 453,012 | 100.0 |  | 67,244 | 100.0 |  | 20,627 | 100.0 |  | 30.7 | |
| Berlin (Nord, Mitte, Süd) | Cities | 2,365,777 | 91.3 |  | 255,003 | 84.2 |  | 25,120 | 80.5 |  | 9.9 | |
|  | Towns and suburbs | 209,014 | 8.1 |  | 43,139 | 14.3 |  | 5,407 | 17.3 |  | 12.5 | |
|  | Rural areas | 15,828 | 0.6 |  | 4,550 | 1.5 |  | 688 | 2.2 |  | 15.1 | |
|  | Total | 2,590,619 | 100.0 |  | 302,692 | 100.0 |  | 31,215 | 100.0 |  | 10.3 | |
| Berlin-Mitte | Cities | N/A^a^ | N/A^a^ |  | 80,810 | 100.0 |  | 11,026 | 100.0 |  | 13.6 | |
|  | Towns and suburbs |  | - |  |  | - |  |  | - |  |  | |
|  | Rural areas |  | - |  |  | - |  |  | - |  |  | |
|  | Total | N/A^a^ | 100.0 |  | 80,810 | 100.0 |  | 11,026 | 100.0 |  | 13.6 | |
| Berlin-Nord | Cities | N/A^a^ | N/A^a^ |  | 48,786 | 54.2 |  | 4,593 | 45.4 |  | 9.4 | |
|  | Towns and suburbs | 141,904 | N/A^a^ |  | 36,677 | 40.7 |  | 4,844 | 47.8 |  | 13.2 | |
|  | Rural areas | 15,828 | N/A^a^ |  | 4,550 | 5.1 |  | 688 | 6.8 |  | 15.1 | |
|  | Total | N/A^a^ | N/A^a^ |  | 90,013 | 100.0 |  | 10,125 | 100.0 |  | 11.2 | |
| Berlin-Süd | Cities | N/A^a^ | N/A^a^ |  | 125,407 | 95.1 |  | 9,501 | 94.4 |  | 7.6 | |
|  | Towns and suburbs | 67,110 | N/A |  | 6,462 | 4.9 |  | 563 | 5.6 |  | 8.7 | |
|  | Rural areas |  | - |  |  | - |  |  | - |  |  | |
|  | Total | N/A^a^ | N/A^a^ |  | 131,869 | 100.0 |  | 10,064 | 100.0 |  | 7.6 | |
| Bremen | Cities | 368,016 | 100.0 |  | 84,374 | 100.0 |  | 10,484 | 100.0 |  | 12.4 | |
|  | Towns and suburbs |  | - |  |  | - |  |  | - |  |  | |
|  | Rural areas |  | - |  |  | - |  |  | - |  |  | |
|  | Total | 368,016 | 100.0 |  | 84,374 | 100.0 |  | 10,484 | 100.0 |  | 12.4 | |
| Düsseldorf | Cities | 410,503 | 100.0 |  | 73,974 | 100.0 |  | 9,153 | 100.0 |  | 12.4 | |
|  | Towns and suburbs |  | - |  |  | - |  |  | - |  |  | |
|  | Rural areas |  | - |  |  | - |  |  | - |  |  | |
|  | Total | 410,503 | 100.0 |  | 73,974 | 100.0 |  | 9,153 | 100.0 |  | 12.4 | |
| Essen | Cities | 379,084 | 100.0 |  | 60,810 | 100.0 |  | 10,660 | 100.0 |  | 17.5 | |
|  | Towns and suburbs |  | - |  |  | - |  |  | - |  |  | |
|  | Rural areas |  | - |  |  | - |  |  | - |  |  | |
|  | Total | 379,084 | 100.0 |  | 60,810 | 100.0 |  | 10,660 | 100.0 |  | 17.5 | |
| Freiburg | Cities | 154,615 | 57.9 |  | 32,959 | 58.4 |  | 6,061 | 60.0 |  | 18.4 | |
|  | Towns and suburbs | 89,008 | 33.4 |  | 18,683 | 33.1 |  | 3,226 | 31.9 |  | 17.3 | |
|  | Rural areas | 23,246 | 8.7 |  | 4,781 | 8.5 |  | 818 | 8.1 |  | 17.1 | |
|  | Total | 266,869 | 100.0 |  | 56,423 | 100.0 |  | 10,105 | 100.0 |  | 17.9 | |
| Halle | Cities | 152,944 | 81.6 |  | 31,635 | 74.3 |  | 7,622 | 75.2 |  | 24.1 | |
|  | Towns and suburbs | 7,167 | 3.8 |  | 2,283 | 5.4 |  | 515 | 5.1 |  | 22.6 | |
|  | Rural areas | 27,257 | 14.5 |  | 8,677 | 20.4 |  | 2,004 | 19.8 |  | 23.1 | |
|  | Total | 187,368 | 100.0 |  | 42,595 | 100.0 |  | 10,141 | 100.0 |  | 23.8 | |
| Hamburg | Cities | 1,195,458 | 100.0 |  | 64,149 | 100.0 |  | 10,112 | 100.0 |  | 15.8 | |
|  | Towns and suburbs |  | - |  |  | - |  |  | - |  |  | |
|  | Rural areas |  | - |  |  | - |  |  | - |  |  | |
|  | Total | 1,195,458 | 100.0 |  | 64,149 | 100.0 |  | 10,112 | 100.0 |  | 15.8 | |
| Hannover | Cities | 356,686 | 100.0 |  | 82,064 | 100.0 |  | 10,065 | 100.0 |  | 12.3 | |
|  | Towns and suburbs |  | - |  |  | - |  |  | - |  |  | |
|  | Rural areas |  | - |  |  | - |  |  | - |  |  | |
|  | Total | 356,686 | 100.0 |  | 82,064 | 100.0 |  | 10,065 | 100.0 |  | 12.3 | |
| Kiel | Cities | 219,135 | 61.7 |  | 37,294 | 67.4 |  | 6,185 | 65.1 |  | 16.6 | |
|  | Towns and suburbs | 98,178 | 27.6 |  | 13,277 | 24.0 |  | 2,349 | 24.7 |  | 17.7 | |
|  | Rural areas | 38,134 | 10.7 |  | 4,772 | 8.6 |  | 974 | 10.2 |  | 20.4 | |
|  | Total | 355,447 | 100.0 |  | 55,343 | 100.0 |  | 9,508 | 100.0 |  | 17.2 | |
| Leipzig | Cities | 364,325 | 85.1 |  | 37,164 | 76.3 |  | 8,760 | 80.6 |  | 23.6 | |
|  | Towns and suburbs | 54,960 | 12.8 |  | 10,169 | 20.9 |  | 1,861 | 17.1 |  | 18.3 | |
|  | Rural areas | 8,862 | 2.1 |  | 1,381 | 2.8 |  | 250 | 2.3 |  | 18.1 | |
|  | Total | 428,147 | 100.0 |  | 48,714 | 100.0 |  | 10,871 | 100.0 |  | 22.3 | |
| Mannheim | Cities | 205,066 | 100.0 |  | 79,574 | 100.0 |  | 10,299 | 100.0 |  | 12.9 | |
|  | Towns and suburbs |  | - |  |  | - |  |  | - |  |  | |
|  | Rural areas |  | - |  |  | - |  |  | - |  |  | |
|  | Total | 205,066 | 100.0 |  | 79,574 | 100.0 |  | 10,299 | 100.0 |  | 12.9 | |
| Münster | Cities | 210,001 | 100.0 |  | 56,313 | 100.0 |  | 10,042 | 100.0 |  | 17.8 | |
|  | Towns and suburbs |  | - |  |  | - |  |  | - |  |  | |
|  | Rural areas |  | - |  |  | - |  |  | - |  |  | |
|  | Total | 210,001 | 100.0 |  | 56,313 | 100.0 |  | 10,042 | 100.0 |  | 17.8 | |
| Neubrandenburg | Cities | 45,834 | 23.2 |  | 21,977 | 20.9 |  | 5,575 | 25.3 |  | 25.4 | |
|  | Towns and suburbs | 47,323 | 24.0 |  | 29,034 | 27.6 |  | 5,347 | 24.3 |  | 18.4 | |
|  | Rural areas | 104,325 | 52.8 |  | 54,338 | 51.6 |  | 11,127 | 50.5 |  | 20.5 | |
|  | Total | 197,482 | 100.0 |  | 105,349 | 100.0 |  | 22,049 | 100.0 |  | 20.9 | |
| Regensburg | Cities | 99,989 | 43.7 |  | 17,660 | 38.0 |  | 3,361 | 33.5 |  | 19.0 | |
|  | Towns and suburbs | 68,966 | 30.2 |  | 13,141 | 28.3 |  | 2,870 | 28.6 |  | 21.8 | |
|  | Rural areas | 59,770 | 26.1 |  | 15,663 | 33.7 |  | 3,803 | 37.9 |  | 24.3 | |
|  | Total | 228,725 | 100.0 |  | 46,464 | 100.0 |  | 10,034 | 100.0 |  | 21.6 | |
| Saarbrücken | Cities | 129,266 | 38.4 |  | 33,025 | 36.7 |  | 3,800 | 37.8 |  | 11.5 | |
|  | Towns and suburbs | 188,266 | 55.9 |  | 51,473 | 57.2 |  | 5,502 | 54.8 |  | 10.7 | |
|  | Rural areas | 19,413 | 5.8 |  | 5,475 | 6.1 |  | 747 | 7.4 |  | 13.6 | |
|  | Total | 336,945 | 100.0 |  | 89,973 | 100.0 |  | 10,049 | 100.0 |  | 11.2 | |
| NAKO overall | Cities | 336,945 | 83.8 |  | 995,902 | 75.7 |  | 144,541 | 70.4 |  | 14.5 |  |
|  | Towns and suburbs | 6,847,133 | 11.5 |  | 207,853 | 15.8 |  | 35,831 | 17.4 |  | 17.2 |  |
|  | Rural areas | 936,025 | 4.7 |  | 112,300 | 8.5 |  | 25,042 | 12.2 |  | 22.3 |  |
|  | Total | 386,270 | 100.0 |  | 1,316,055 | 100.0 |  | 205,414 | 100.0 |  | 15.6 |  |

^a^ Study base characteristics are not available for the three individual study centers in the region Berlin because all three centers jointly recruited within the city of Berlin.

**Supplementary Figures**

**Figure S1** Response proportion (%) and frequency of non-mandatory recruitment steps. (a) Overall response in study centers by percentage of outbound calls and number of reminders. Outbound calls refer to call attempts documented prior to an active response from the invited individual occurred. The pattern of the scatter plot suggests that an increased percentage of outbound calls is associated with a higher response, regardless of whether a study center routinely sent out 2 reminder letters (red dots) or 3 reminder letters (turquoise dots). (b) Box plots comparing overall response across study centers using 2 and 3 reminders. The number of reminder letters did not seem to result in different response proportions. (c) Box plots comparing overall response across study centers below and above the median of outbound calls (3%). Study centers that made more outbound calls achieved higher response proportions than with less outbound call attempts.


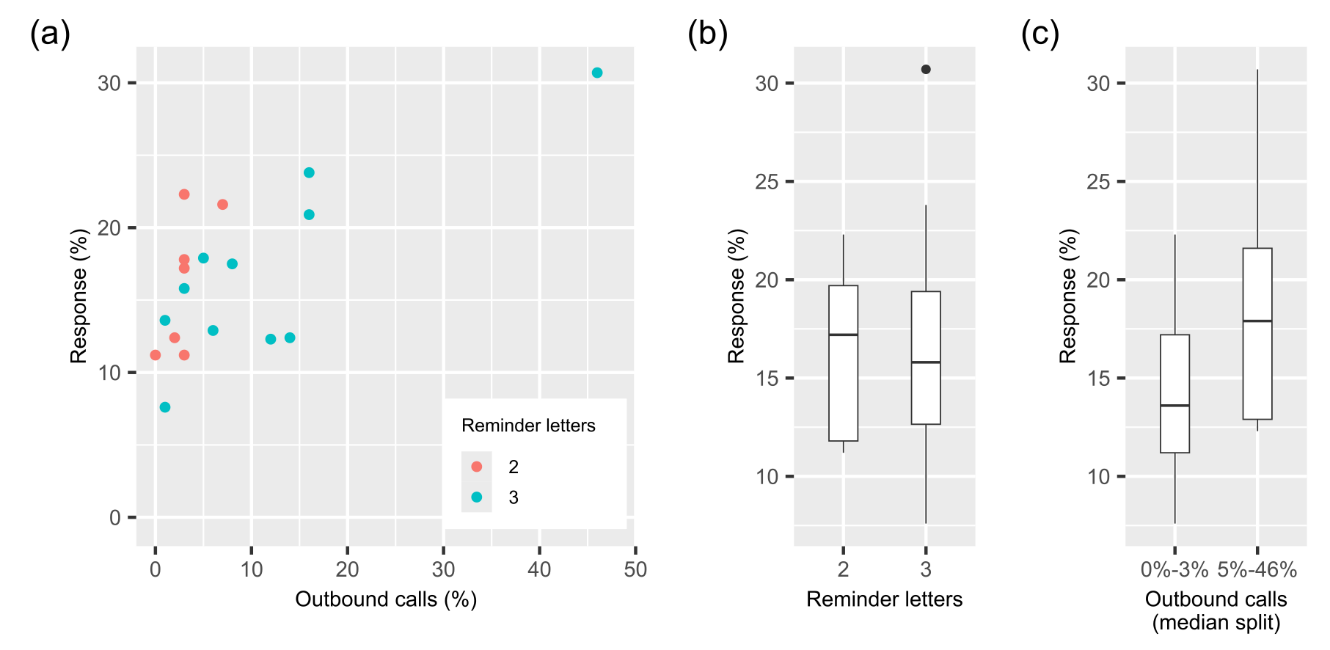


**Figure S2** Comparision of the unweighted and weighted samples for 16 NAKO study regions with respect to sex, age group, nationality, migration background, education, and household size


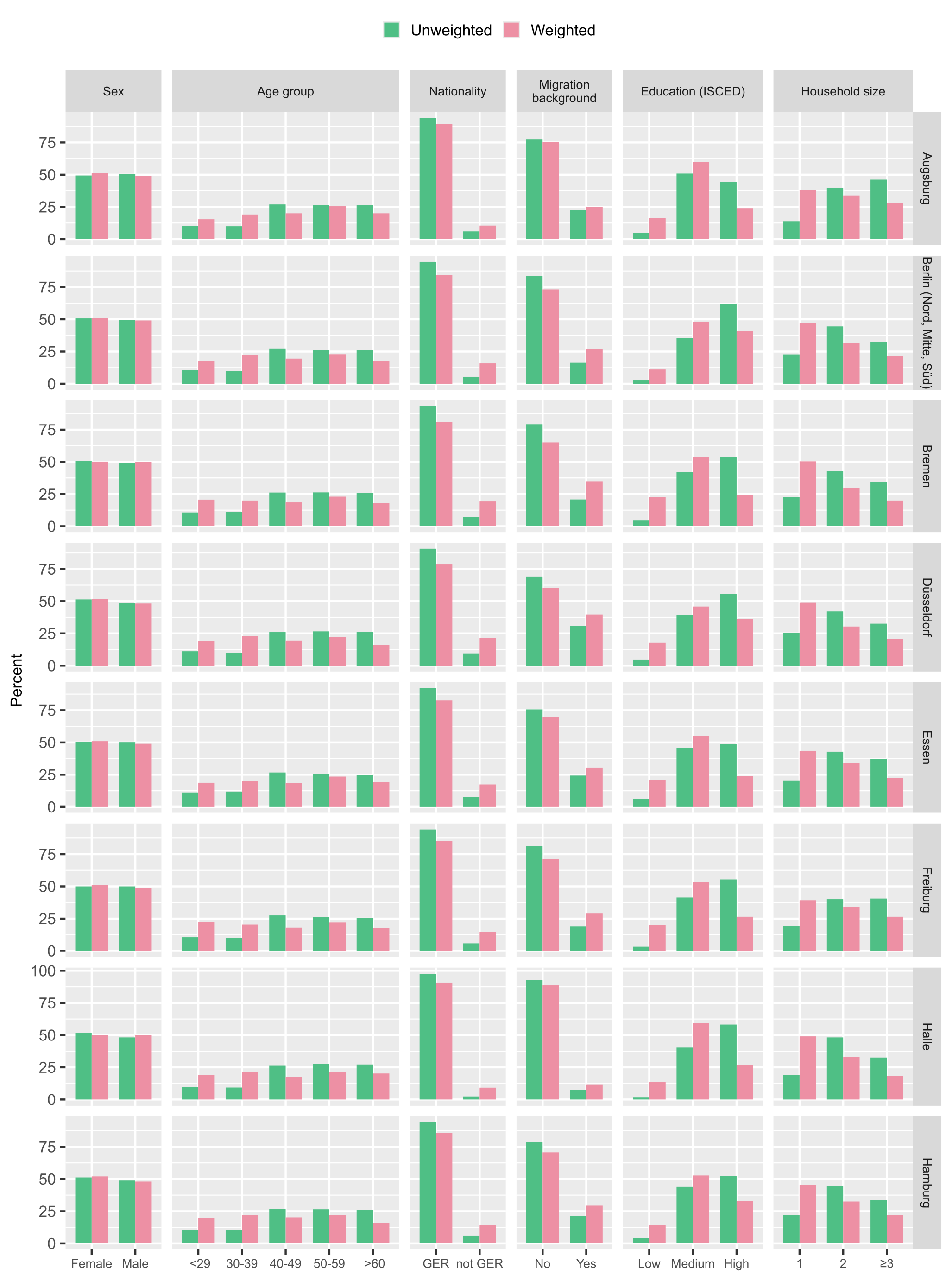


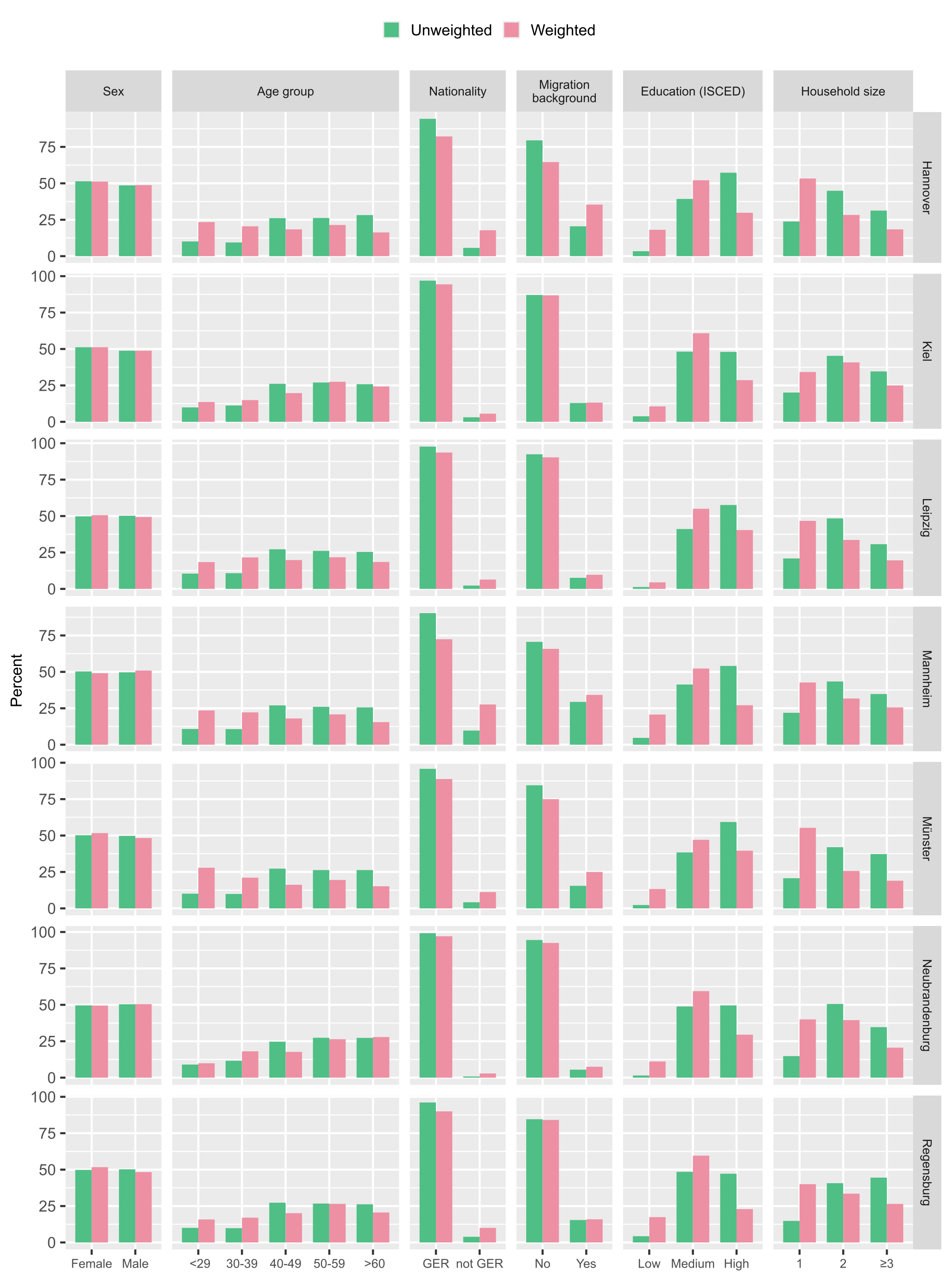

Supplement: Supplementary file 1 — Supplementary file1 (DOCX 628 KB) [file 10654_2025_1219_MOESM1_ESM.docx]
